# A new member of the fusaricidin family – structure elucidation and synthesis of fusaricidin E

**DOI:** 10.3762/bjoc.13.140

**Published:** 2017-07-20

**Authors:** Marcel Reimann, Louis P Sandjo, Luis Antelo, Eckhard Thines, Isabella Siepe, Till Opatz

**Affiliations:** 1 Institute of Organic Chemistry, Johannes Gutenberg-University, Duesbergweg 10–14, 55128 Mainz, Germany; 2 Departamento de Ciências Farmacêuticas, Centro de Ciências da Saúde, Bloco J/K, Universidade Federal de Santa Catarina, Florianópolis 88040-900, SC, Brazil; 3 Institute of Biotechnology and Drug Research, Erwin Schrödinger-Str. 56, 66776 Kaiserslautern, Germany; 4 Institute of Molecular Physiology, Microbiology and Wine Research, Johannes Gutenberg University Mainz, Johann-Joachim-Becher-Weg 15, 55128 Mainz, Germany; 5 BASF SE, 67056 Ludwigshafen, Germany

**Keywords:** cyclodepsipeptides, fusaricidins, lipopeptides, structure elucidation, total synthesis

## Abstract

Two hitherto unknown fusaricidins were obtained from fermentation broths of three *Paenibacillus* strains. After structure elucidation based on tandem mass spectrometry and NMR spectroscopy, fusaricidin E was synthesized to confirm the structure and the suggested stereochemistry. The synthesis was based on a new strategy which includes an efficient access to the 15-guanidino-3-hydroxypentadecanoyl (GHPD) side chain from erucamide.

## Introduction

Fusaricidins are lipid-modified non-ribosomal cyclic hexadepsipeptides containing four D-amino acids and two L-amino acids. All of them carry an L-threonine linked to a unique 15-guanidino-3-hydroxypentadecanoic acid side chain through the *N*-terminus. This particular ω-functionalized lipid side chain is of key importance for the antibiotic activity of the fusaricidins and their selective inhibition of bacterial cells due to the interaction with phospholipid cell membranes [1]. Genetic analysis of the producing organisms suggests that the biosynthesis of this essential part of the fusaricidins most likely shows similarity to the fatty acid synthesis pathway [2].

All fusaricidins have three amino acids (L-Thr, D-*allo-*Thr, and D-Ala) in common and are mostly isolated in pairs which differ in a single amino acid (asparagine vs glutamine). There are several known members of the fusaricidin family which were isolated from several strains of *Paenibacillus polymyxa* [3–4]. Fusaricidins exhibit antimicrobial activity against Gram-positive bacteria and a wide range of fungi including *Leptosphaeria maculans*, a plant pathogenic fungus responsible for the blackleg disease on *Brassica* crops [5–7].

**Figure 1:**
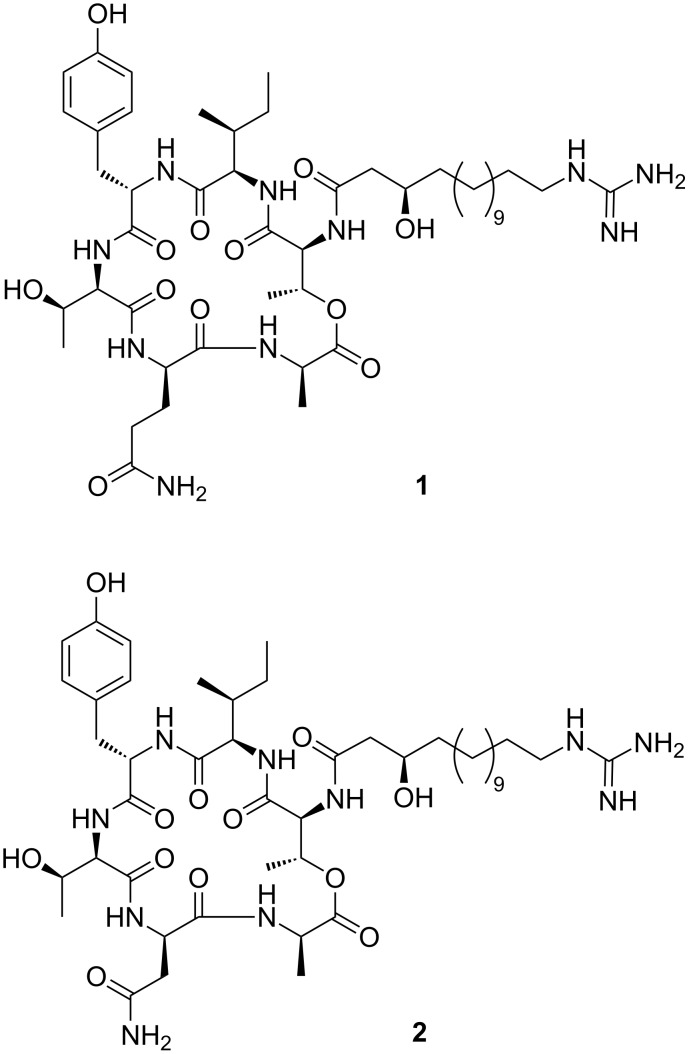
Structure of fusaricidins E (**1**) and F (**2**).

Two new compounds were obtained from fermentation of *Paenibacillus sp.* strain Lu16774 as an inseparable mixture of two homologous cyclic depsipeptides containing either glutamine (in **1**) or asparagine (in **2**). Initially, the absolute and relative configuration of the isoleucine residue was unknown (Figure 1). Thus, an assumed stereoisomer (containing D-*allo-*Ile) of the new fusaricidin member was synthesized based on analogy to known members of the series and compared to the natural product [8].

## Results and Discussion

### Isolation and structure elucidation

The *Paenibacillus* strain was cultivated on agar plates containing GYM medium (10 g/L glucose, 4 g/L yeast extract, 10 g/L malt extract; pH 5.5, adjusted before autoclaving) and 20 g/L agar. The submerged cultivation was carried out for 10 to 20 days at room temperature. For maintenance, agar slants containing the same medium were used and stored at 4 °C. Small scale liquid cultures (250 mL GYM medium in 500 mL flasks) were inoculated with 4–5 pieces of a well grown agar culture and cultivated on an orbital shaker at 120 rpm at room temperature. Large scale fermentations were carried out in 20 L fermenters with 15 L GYM medium inoculated with 250 mL well grown liquid culture at room temperature with agitation (120 rpm) and aeration (3 L/min) for 5 to 8 days. To prevent excessive foaming, a few mL of silicone antifoam were added prior to sterilization of the medium.

An equal volume of isopropanol was added to the culture. After agitation for 2 h, 200 g/L sodium chloride was added to the mixture until phase separation of the organic and aqueous phase was visible. The isopropanol layer was concentrated in vacuo. The resulting extract was dissolved in methanol, centrifuged for better precipitation of salt residues, and the organic phase was concentrated in vacuo again. This step was repeated until no further salt precipitates were visible.

A portion of the extract (30 g) was dissolved in methanol and adsorbed to silica gel (Merck, K60, 70–230 mesh, 50 g), dried at 40 °C and applied onto silica gel (1 kg, column 10 cm diameter, 30 cm height). Elution was carried out in four steps as follows: ethyl acetate, ethyl acetate/methanol (3:1, v/v), ethyl acetate/methanol (1:1, v/v) and methanol. The third fraction containing the active compounds, was dried in vacuo and dissolved in 40% methanol (MeOH) in 0.1% formic acid (FA, concentration: 100 mg/mL). The other fractions were discarded. A fraction (20 mL) of the sample was loaded onto a previously equilibrated (with 40% MeOH in 0.1% FA) Chromabond HR-X cartridge (Macherey-Nagel, 1000 mg). The cartridge was washed with 100 mL 40% MeOH in 0.1% FA and eluted with 60 mL 70% MeOH in 0.1% FA. The sample was dissolved in DMSO (concentration: 200 mg/mL) and 300 μL thereof were applied to a Sunfire C18 column (19 × 250 mm, 5 μm, Waters) and eluted as follows: 16 min at 10 mL/min, isocratic 70% 0.2% FA; 30% acetonitrile (MeCN), 1 min at 14 mL/min, gradient to 65% 0.2% FA; 35% MeCN, 5 min at 14 mL/min, isocratic 65% 0.2% FA; 35% MeCN. The five resulting fractions were dried in vacuo and re-dissolved in DMSO (concentration: 125 mg/mL). Further purification was performed using the same column and isocratic conditions (flow: 10.5 mL/min) adjusted for every fraction (12.5 mg per run). In the second fraction 68% 0.2% FA; 32% MeCN was used and a single peak was detected, consisting of an inseparable mixture of two novel fusaricidins which were named fusaricidin E (**1**) and fusaricidin F (**2**). The mixture (6.0 mg) was composed of 3 parts of **2** and 7 parts of its higher homologue **1** and had an optical rotation of [α]_D_^25^ +20.9 (*c* 0.6, DMSO-*d*_6_). The mass difference between both metabolites is 14 amu. This observation was supported by two ion peaks observed in the ESIMS spectrum at *m/z* 961.6 and *m*/*z* 975.6, respectively.

**Figure 2:**
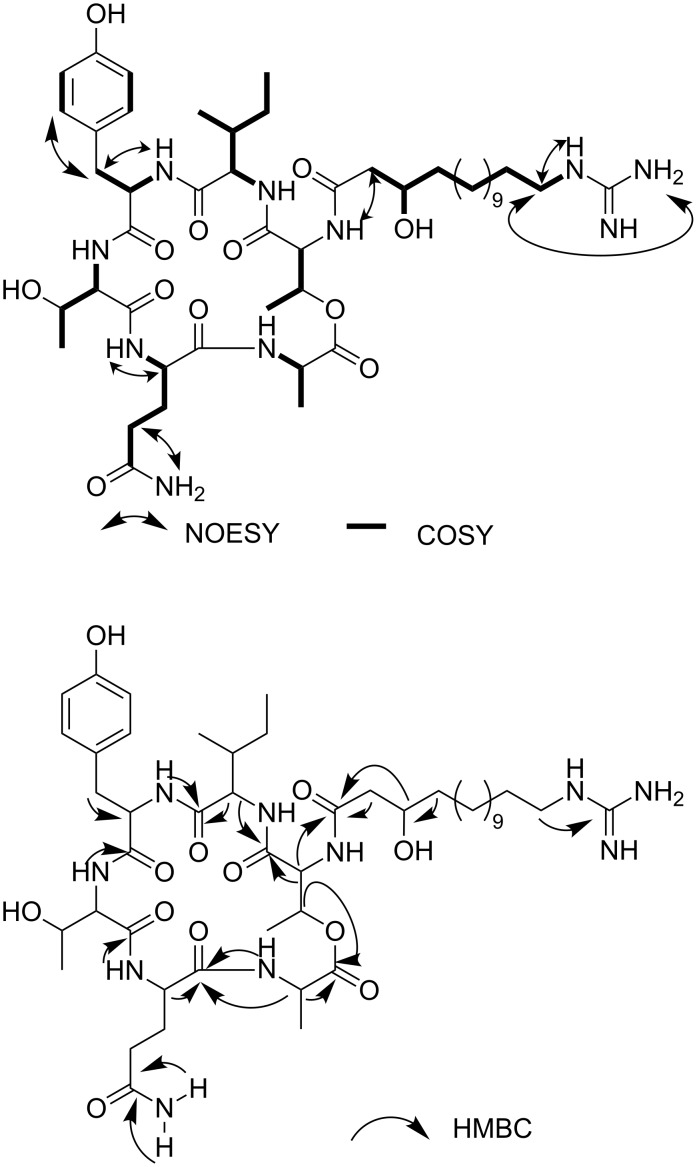
NOESY /COSY and HMBC correlations of compound **1**.

Through extensive analysis of the 1 and 2D NMR data of the major component **1**, six amino acids including tyrosine (Tyr), glutamine (Gln), alanine (Ala), two threonines (Thr1 and Thr2) and isoleucine (Ile) were identified. NMR experiments like COSY, NOESY and HMBC (Figure 2) clarified the sequence of the amino acids through two or three bonds correlation.

**Figure 3:**
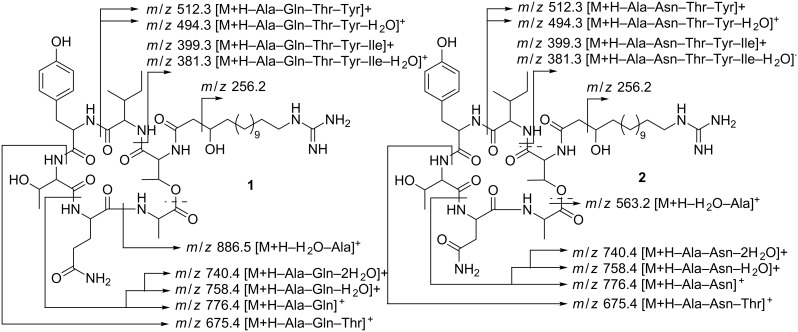
Fragmentation pattern of compounds **1** and **2**.

The spectra revealed correlations from the Thr2 NH at δ 8.50 to the α-CH of Thr2 at δ 3.94 and the carbonyl of Tyr at δ 166.7. The same correlations were found for the Tyr NH at δ 8.52, the Tyr α-CH at δ 2.60 and the carbonyl of Ile at δ 170.4. The spectra also showed a strong correlation of the Ile α-CH at δ 4.16 with the carbonyl signal of Ile at δ 170.4 and a weak correlation with the carbonyl of Thr1 at δ 168.6. Furthermore, the β-methine proton at δ 5.30 of Thr1 correlated with the carbonyl signal at δ 170.4 of Ala and additionally the NH of Ala at δ 7.27 had the expected contact with Ala’s α-CH at δ 4.20. The latter proton showed correlations with the carbonyl of the same amino acid and the one of Gln. The NH proton of Gln at δ 8.20 displayed correlations with the methine hydrogen of Gln at δ 3.87 and the carbonyl of Thr2 at δ 170.6. All data suggested a cyclodepsipeptidic structure for **1**. The N-atom of Thr1 was bound to a guanidine β-hydroxy fatty acid as a key correlation was observed between the signal of its α-methine proton at δ 4.46 and the resonance of a carbonyl at δ 171.9. This carbonyl showed HMBC correlation with α-methylene protons at δ 2.35 and the β-methine proton at δ 3.77. Additionally correlations between methylene protons at δ 3.03 and the guanidine carbon at δ 157.2 were found. The length of the side chain between the β-hydroxy and the guanidine group was affirmed by the fragment ion observed in the APCI–MS–MS spectrum of the parent [M + H]^+^ ion at *m*/*z* 256.2. Likewise, this spectrum provided information (Figure 3) which confirmed the connection sequence of amino acids and led to elucidate the structure of **1** and **2** as shown below.

Signals of geminal hydrogen atoms at δ 2.80, 2.52 revealed a direct correlation with the carbon atom δ 36.3 in the HSQC spectrum. Based on long-range H–H and H–C interactions, these resonances were assigned to the β-CH_2_ group of asparagine (Asn) in the lower homologue **2**. Thus, the conclusion was supported by reported data for Asn in other fusaricidins [9] in conjunction to fragments obtained from the tandem mass of the parent peak at *m*/*z* 961.6 (Figure 3).

### Retrosynthetic plan

For our synthesis, a novel and efficient access to the GHPD side chain starting from erucamide (**6**) as an inexpensive natural source for the required C_13_-fragment was developed. After ozonolysis of the Fmoc-protected amine obtained by reduction and alkoxycarbonylation from **6**, the stereocenter should be generated by nucleophilic addition of an allyl anion equivalent to the resulting aldehyde **5**.

**Scheme 1:**
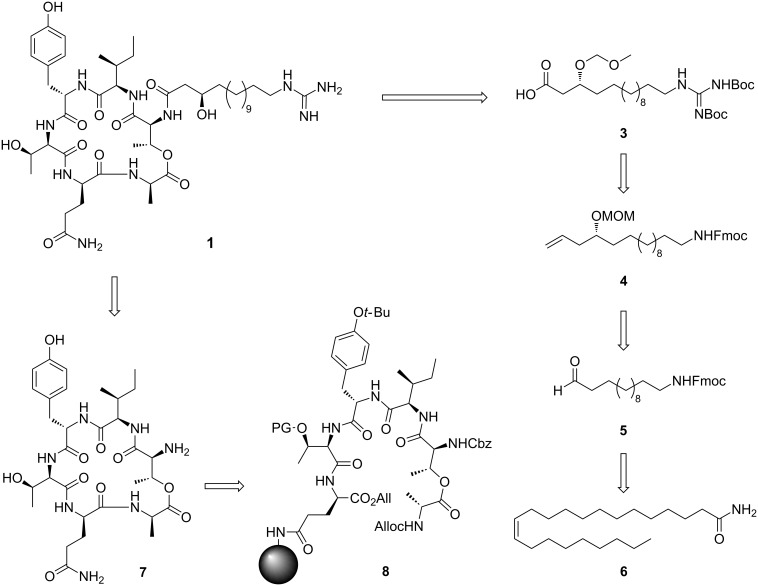
Retrosynthetic plan for the depsipeptide and GHPD side chain.

Guanidine formation and ozonolysis with subsequent oxidation to the carboxylic acid would then furnish the protected GHPD side chain building block **3** which can then be coupled to the cyclodepsipeptide fragment to give the desired product **1** (Scheme 1). For the synthesis of the cyclodepsipeptide portion, a convergent route was envisaged in which the complete GHPD side chain should be attached in solution after assembly of the cyclodepsipeptide on solid support. The hitherto only published synthesis of a natural fusaricidin by the Jolliffe group employed a ring closure via a lactonization in solution and subsequent attachment of the side chain to the cyclized depsipeptide [10]. Since the macrolactonization approach suffered from diastereoselectivity issues and low yield, it was decided to perform an on-resin head-to-tail macrolactamization instead [11]. A similar strategy had been used by Cudic and co-workers to synthesize analogs of fusaricidin A with an on resin coupling of a 12-aminododecanoic acid combined with a late stage guanidinylation to give the unnatural 12-guanidinyldodecanoic acid side chain [12]. In order to reduce the number of linear steps, the protected guanidino group was included in the side chain building block in our case. This strategy would allow to assemble the complete peptide core in a solid-phase synthesis and to perform the solution-phase coupling without a large excess of the GHPD side chain building block. Thus, Cudic’s SPPS approach should be combined with the advantages of the late stage coupling employed by Jolliffe.

### Synthesis

**Scheme 2:**
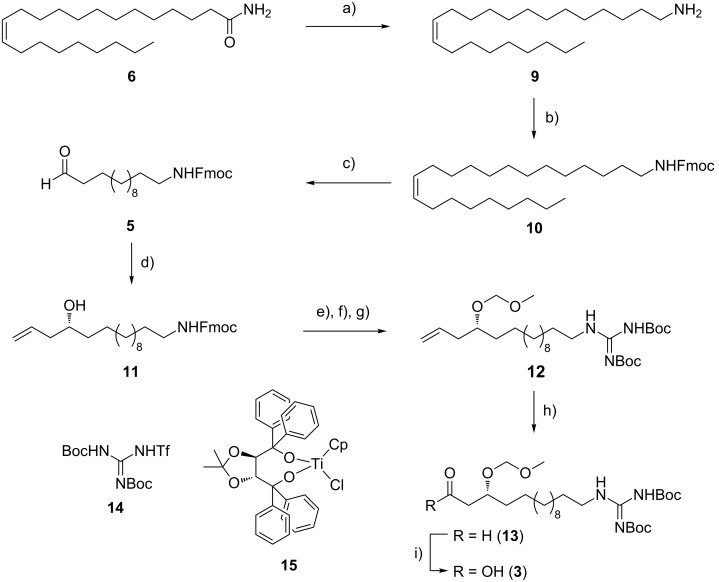
a) LiAlH_4_, THF, reflux, 12 h, quant.; b) Fmoc-OSu, NaHCO_3_, 1,4-dioxane, H_2_O, 0 °C to rt, 87%; c) 1: O_3_, DCM, –78 °C, 2: Zn, HOAc, –78 °C to 10 °C, 66%; d) allylmagnesium chloride, **15**, Et_2_O, –78 °C, 84%, 94% ee; e) MOMCl, DIPEA, DCM 0 °C to rt; f) piperidine, DCM, rt; g) **14**, NEt_3_, DCM, 49% (over 3 steps); h) 1: O_3_, DCM, –78 °C, 2: PPh_3_, –78 °C → rt, 77%; i) NaClO_2_, NaH_2_PO_4_, amylene, *t*-BuOH, H_2_O, rt, 80%.

The C_13_-fragment was prepared starting from erucamide (**6**) in three simple operations. Reduction of the amide with lithium aluminium hydride, followed by Fmoc-protection and ozonolysis furnished aldehyde **5** in 57% yield over three steps (Scheme 2).

The homoallylic alcohol was prepared by an enantioselective Duthaler–Hafner allylation [13] with the titanium-complex **15** in high yield and 94% ee. Attempts to perform a catalytic Keck allylation with BINOL-titanium catalysts failed due to low conversion [14]; however, in spite of good experience with the Maruoka–Keck allylation in another total synthesis, the conversion was not satisfying in this case [15]. After protection and guanidinylation with triflylguanidine **14**, the homoallylic alcohol **12** was subjected to ozonolysis and Pinnick oxidation to furnish the protected GHPD acid **3** in an overall yield of 14.3%.

**Scheme 3:**
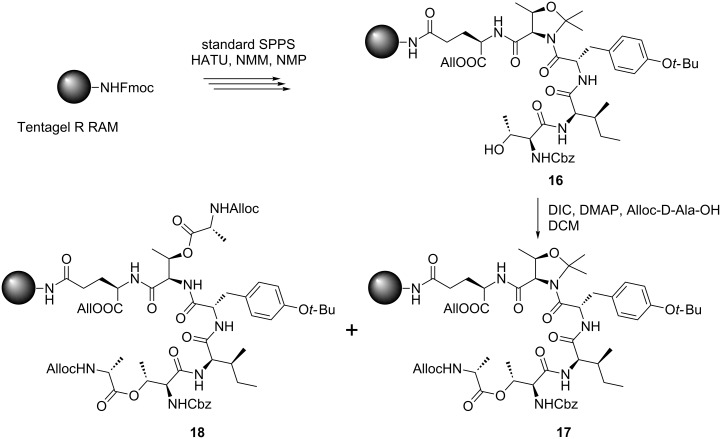
Ester bond formation with 2,2-dimethylated pseudoproline including peptide **16**.

The peptide core was synthesized manually according to a standard SPPS Fmoc protocol using HATU and NMM in NMP [16]. For protection of the threonine unit, it was converted into a 2,2-dimethylated pseudoproline (Ψ^Me,Me'^Pro). This method has been reported to improve yields of macrolactamizations through stabilizing the *cis*-configuration of the amide bond and working as a turn inducer in peptides [17–20]. The Ψ^Me,Me'^Pro unit turned out not to be completely stable during the esterification with DIC/DMAP [12] as the double acylation product **18** could be detected (Scheme 3).

Further investigations suggested DMAP to be responsible for the cleavage of the Ψ^Me,Me'^Pro and after reduction of the amount of DMAP to 5 mol %, nearly no doubly acylated product was found.

**Scheme 4:**
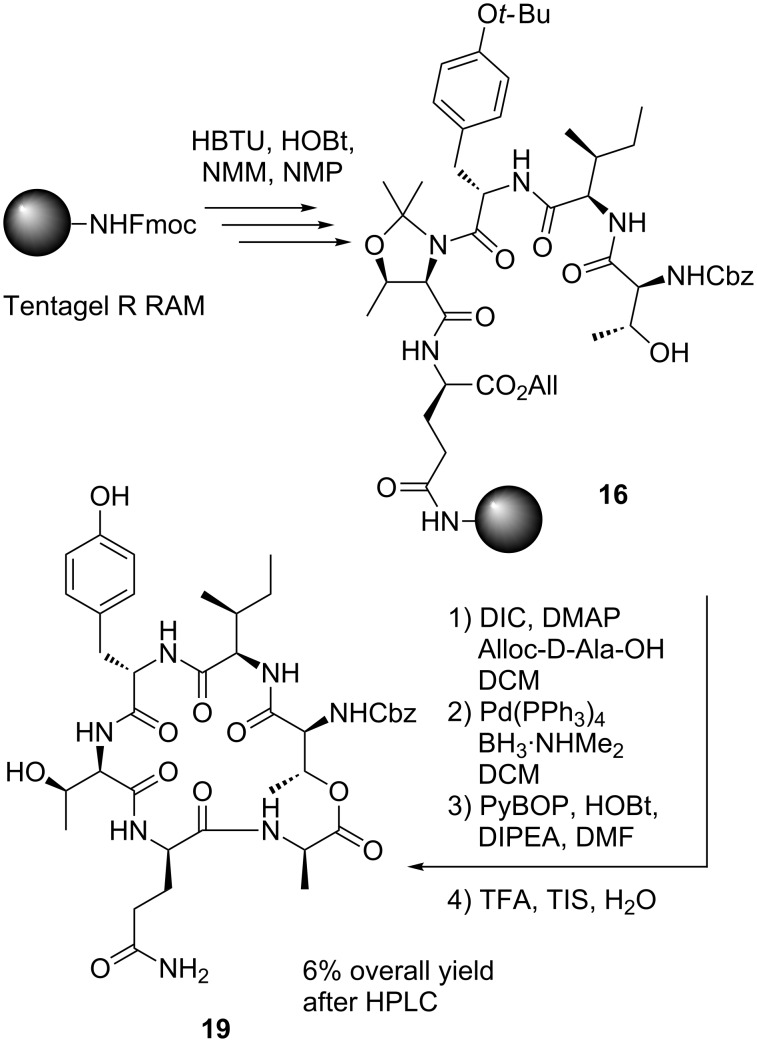
Cyclization with 2,2-dimethylated pseudoproline including peptide **16**.

Besides the doubly acylated product, several other uncharacterized byproducts were formed. Nevertheless, *O*-deallylation with Pd(PPh_3_)_4_ and BH_3_·NHMe_2_ gave a high conversion to the deprotected peptide [1]. The cyclization with PyBOP, HOBt and DIPEA in DMF led again to the formation of numerous byproducts. In contrast to literature reports [12], it was found that cleavage from the resin with “reagent K” [12,21] was inferior to a TFA/TIS/H_2_O mixture (95:2.5:2.5). Due to the instability of Ψ^Me,Me'^Pro during esterification and cyclization, the isolated yield of **19** was only 6% (Scheme 4).

**Scheme 5:**
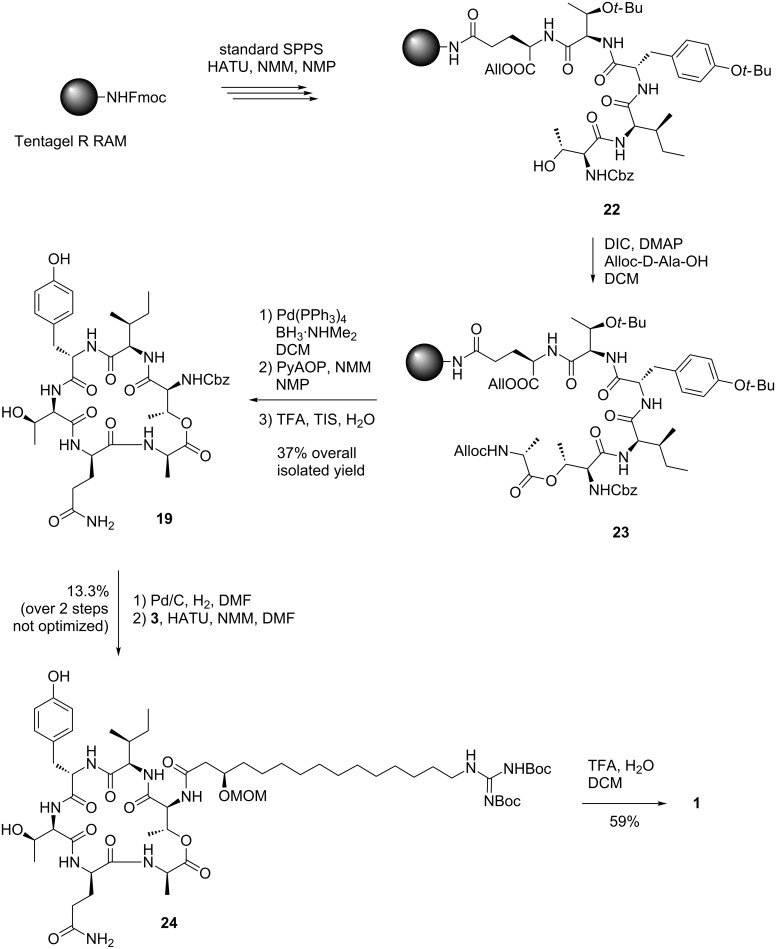
Depsipeptide cyclization and coupling with GHPD side chain.

To improve this, O-*tert*-butyl-protected D-*allo-*Thr was employed instead. For the O-*tert-*butyl-protected D-*allo-*Thr, clean and complete conversion was observed during both esterification and deprotection. This time, cyclization was performed with PyAOP and NMM in NMP to provide **19** in an isolated yield of 37% (Scheme 5).

Unfortunately, we observed two signal sets in NMR spectra (intensity 3:1) for the product which could not be attributed to conformers or rotamers as evidenced by variable temperature and NOESY NMR experiments.

These results, and the fact that Cochrane also reported problems due to epimerization during macrolactonization suggested that a partial loss of stereochemical integrity had taken place during either cyclization or esterification [11]. The site of epimerization could not be determined with certainty, but most likely, the D-Ala residue was affected.

**Figure 4:**
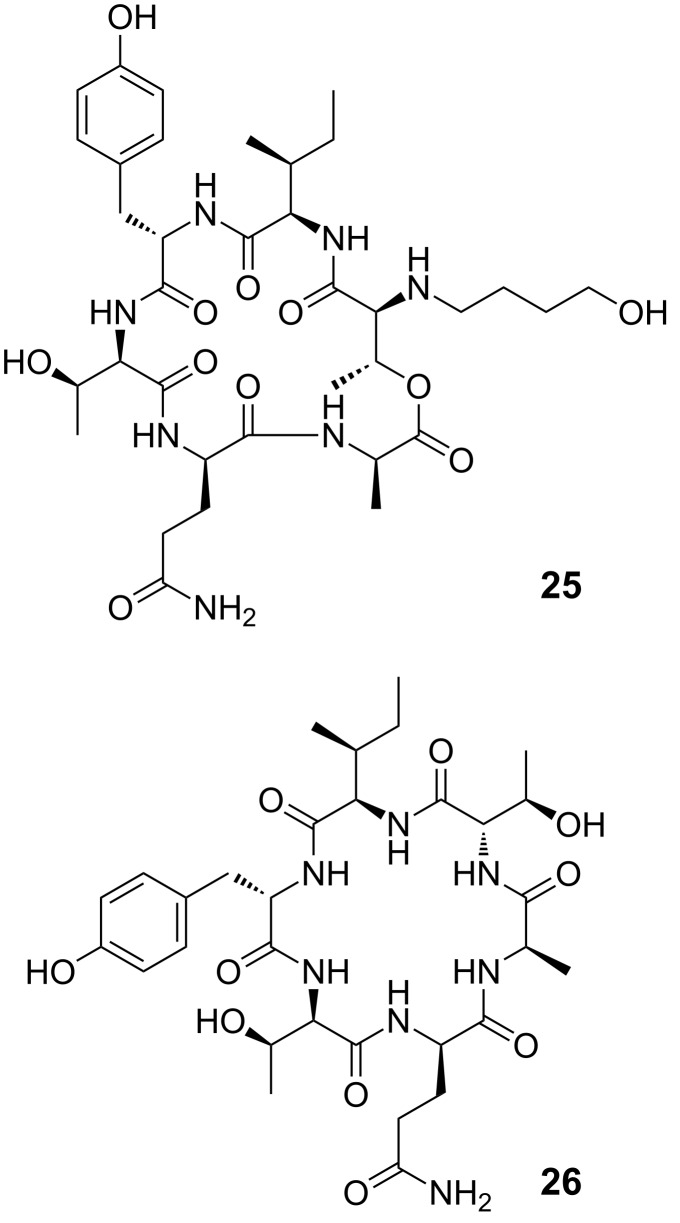
Byproducts from removal of Cbz group in THF and DMF.

As the assumed diastereomers could not be observed or separated by HPLC, the next steps were performed with the mixture. Performing the removal of the Cbz group with H_2_/Pd-C in THF, we encountered the formation of the *N*-(4-hydroxybutylated) product **25** resulting from a ring opening reaction of the solvent (Figure 4). This side reaction has been reported for unstabilized THF [22] while stabilized THF was used in our case.

Due to solubility problems in 1,4-dioxane and other solvents suitable for hydrogenolysis, the deprotection was thus performed in DMF [23–24]. Unfortunately, an *O→N* acyl shift to product **26** could be observed as judged by NMR spectroscopy when aged DMF was used for this purpose. In fresh DMF, hydrogenolysis smoothly produced amine **7** instead. When the crude peptide was coupled to the GHPD side chain unit **3**, HPLC showed only a single peak with the correct *m/z* ratio. This compound **24** was isolated by preparative HPLC and NMR analysis showed a single signal set, so the fate of the assumed minor stereoisomer remains unclear and it was probably lost during HPLC purification. The analysis also revealed that the acylation with the GHPD side chain was selective for the amine and no *O*-acylated product was formed. Side chain protecting groups were removed to yield the natural product **1**. HPLC analysis showed only a single peak with the same retention time, mass and fragmentation pattern as the natural product. After purification by preparative HPLC, NMR spectroscopy confirmed that the assumed structure and stereochemistry of the natural product was correct (see pages S38 and S39 of Supporting Information File 1).

## Conclusion

In summary, two new members of the fusaricidin family, fusaricidins E and F, were isolated from fermentation broths of *Paenibacillus sp.* strain Lu16774 as an inseparable mixture of homologs. Structure elucidation of both peptides was performed with extensive NMR spectroscopy and tandem mass spectrometry. The full stereostructure of the major component, fusaricidin E, could be confirmed by total synthesis. It included a macrolactamization approach combined with a late stage attachment of the GHPD side chain which was synthesized by a newly developed and efficient sequence starting from erucamide. Compared to the Jolliffe strategy, yields were improved and the number of solution phase transformations was reduced. On the other hand, the yields of the Cudic synthesis could not be reached and more steps required purification of the respective products.

## Supporting Information

File 1 Procedures for the synthesis and characterisation data of the compounds.
