# Supplementary material for: A new member of the fusaricidin family – structure elucidation and synthesis of fusaricidin E
Source: Beilstein J Org Chem. 2017 Jul 20;13:1430–8. doi: 10.3762/bjoc.13.140 (PMC5530608; doi:10.3762/bjoc.13.140)
Supplement: File 1 — Procedures for the synthesis and characterisation data of the compounds. [file Beilstein_J_Org_Chem-13-1430-s001.pdf]

# Supporting Information

**for**

# A new member of the fusaricidin family – structure elucidation and synthesis of fusaricidin E

Marcel Reimann[1], Louis P. Sandjo[1,2], Luis Antelo[3], Eckhard Thines[3,4], Isabella Siepe[5] and Till Opatz[1,*]

Address: [1]Institute of Organic Chemistry, Johannes Gutenberg-University, Duesbergweg 10–14, 55128 Mainz, Germany [2]Departamento de Ciências Farmacêuticas, Centro de Ciências da Saúde, Bloco J/K, Universidade Federal de Santa Catarina, Florianópolis 88040-900, SC, Brazil, [3]Institute of Biotechnology and Drug Research, Erwin Schrödinger-Str. 56, 66776 Kaiserslautern, Germany, [4]Institute of Molecular Physiology, Microbiology and Wine Research, Johannes Gutenberg University Mainz, Johann-Joachim-Becher-Weg 15, 55128 Mainz, Germany and [5]BASF SE, 67056 Ludwigshafen, Germany

Email: Till Opatz - opatz@uni-mainz.de

*Corresponding author

**Procedures for the synthesis and characterisation data of the compounds**

## Table of contents

| | | |
|---|---|---|
| I. | NMR-Shift table of natural fusaricidins E and F | S2 |
| II. | Experimental procedures | S5 |
| III. | $^{1}$H and $^{13}$C NMR spectra of compounds | S19 |
| IV. | 2D-NMR spectra of selected compounds | S30 |
| V. | Mosher analysis spectra and selected carbon shifts of cyclopeptide **19** | S37 |
| VI. | NMR-Shift table and $^{13}$C NMR spectra comparison synthetic vs. natural | S38 |
| VII. | References | S39 |

## I) NMR shift table of natural fusaricidins E and F

| Compound 1 | | | Compound 2 | | |
|---|---|---|---|---|---|
| ***Pos.** | **$\delta_H$** | **$\delta_C$** | **Pos.** | **$\delta_H$** | **$\delta_C$** |
| **Thr1** | | | **Thr1** | | |
| NH | 7.79 (br s) | - | NH | 8.18 (br s) | - |
| 1 | - | 168.6 | 1 | - | 168.6 |
| 2 | 4.46 (br d, 8.5) | 56.4 | 2 | 4.39 (br d, 8.7) | 56.9 |
| 3 | 5.30 (m) | 70.2 | 3 | 5.30 (overlapped) | 70.2 |
| 4 | 1.13 (d, 6.4) | 16.7 | 4 | 1.13 (overlapped) | 16.6 |
| **Ala** | | | **Ala** | | |
| NH | 7.27 (br s) | - | NH | 7.22 (br) | - |
| 1 | - | 170.4 | 1 | - | *nf |
| 2 | 4.20 (m) | 47.8 | 2 | 4.13 (overlapped) | 47.7 |
| 3 | 1.17 (d, 7.1) | 17.8 | 3 | 1.11 (overlapped) | 17.8 |
| **Gln** | | | **Asn** | | |
| NH | 8.20 (br s) | - | NH | 8.33 (m) | - |
| 1 | - | 170.4 | 1 | - | 169.7 |
| 2 | 3.87 (m) | 53.2 | 2 | 4.20 (1H, m) | 50.6 |
| 3 | 1.96 (m)<br>2.08 (m) | 26.2 | 3 | 2.52 (overlapped)<br>2.80 (dd, 5.9, 15.1) | 36.3 |
| 4 | 2.08 (m)<br>2.18 (m) | 32.0 | 4 | - | 172.5 |
| - | - | 174.3 | 5 | - | - |
| $NH_2$ | 6.83 (br s),<br>7.26(br s) | - | $NH_2$ | 6.99 (br s)<br>7.42 (br s) | - |
| **Thr2** | | | **Thr2** | | |
| NH | 8.50 (overlapped) | - | NH | 8.58 (br s) | - |
| 1 | - | 170.6 | 1 | - | 170.6 |
| 2 | 3.94 (overlapped) | 59.9 | 2 | 3.84 (m) | 60.5 |

| | | | | | |
|---|---|---|---|---|---|
| 3 | 3.94 (overlapped) | 65.8 | 3 | 3.85 (m) | 65.5 |
| 4 | 1.05 (br) | 20.3 | 4 | 1.08 (overlapped) | 20.0 |
| **Tyr** | | | **Tyr** | | |
| NH | 8.52 (br s) | - | NH | 8.48 (overlapped) | - |
| 1 | - | 166.7 | 1 | - | nf |
| 2 | 4.51 (m) | 54.5 | 2 | 4.60 (m) | 54.2 |
| 3 | 2.60 (m)<br>2.88 (m) | 36.9 | 3 | 2.60 (overlapped)<br>2.88 (overlapped) | 36.8 |
| 4 | - | 127.8 | 4 | - | 127.7 |
| 5 and 9 | 7.06 (d, 8.5) | 130.2 | 5 and 9 | 7.07 (d, 8.7) | 130.2 |
| 6 and 8 | 6.60 (d, 8.5) | 114.7 | 6 and 8 | 6.06 (overlapped) | 114.7 |
| 7 | - | 155.9 | 7 | - | 155.9 |
| **Ile** | | | **Ile** | | |
| NH | 7.42 (br s) | - | NH | 7.28 (br s) | - |
| 1 | - | 170.4 | 1 | - | nf |
| 2 | 4.16 (br d, 8.5) | 56.5 | 2 | 4.16 (overlapped) | 56.5 |
| 3 | 1.34 (m) | 37.2 | 3 | 1.34 (overlapped) | 37.2 |
| 4 | 1.22 (m)<br>1.34 (m) | 25.4 | 4 | 1.34 (overlapped) | 25.4 |
| 5 | 0.53 (overlapped) | 14.4 | 5 | 0.52 (overlapped) | 14.4 |
| 6 | 0.61 (overlapped) | 11.4 | 6 | 0.59 (overlapped) | 11.4 |
| ***FA** | | | **FA** | | |
| 1 | - | 171.9 | 1 | - | 171.9 |
| 2 | 2.35 (m) | 43.3 | 2 | 2.35 (overlapped) | 43.1 |
| 3 | 3.77 (m) | 67.5 | 3 | 3.77 (overlapped) | 67.5 |
| 4 | 1.34 (m) | 36.9 | 4 | 1.34 (overlapped) | 36.8 |
| 5-12 | 1.19-1.30 (br s) | 29.0-29.2 | 5-12 | 1.19-1.30 (br s) | 29.0-29.2 |
| 13 | 1.25 (br s) | 21.2 | 13 | 1.25 (br s) | 21.2 |
| 14 | 1.43 (m) | 28.5 | 14 | 1.43 (overlapped) | 28.7 |

| | | | | | |
|---|---|---|---|---|---|
| 15 | 3.03 (q, 6.6) | 40.6 | 15 | 3.03 (overlapped) | 40.6 |
| ***Gu** | | | **Gu** | | |
| NH | 8.40 (br s) | - | NH | nf | - |
| 16 | - | 157.2 | 16 | - | 157.2 |

*Pos. = position; FA = fatty acid; Gu = Guanidine; nf = not found

# II) Experimental procedures

## General methods

All reagents were reagent grade and used without further purification unless otherwise noted. All reactions involving air or moisture sensitive reagents or intermediates were performed under an inert atmosphere of argon in glassware that was oven dried. Dichloromethane was distilled from $CaH_2$. Degassing of the solvent was performed by three cycles of freeze pump thaw. Reaction temperatures refer to the temperature of the particular cooling/heating bath. Chromatography was performed using flash chromatography of the indicated solvent system on 35–70 µm silica gel (Acros Organics) unless otherwise noted. Alternatively the purifications were performed on an Isolera™ Flash Purification System (Biotage®) with an integrated diode array detector. Reactions were monitored by thin layer chromatography (TLC) carried out on 0.25 mm E. Merck silica gel plates (60F-254) using an aqueous solution of sulfuric acid, $Ce(SO_4)_2{\cdot}4H_2O$ and $(NH_4)_6Mo_7O_{24}{\cdot}4H_2O$ and heat as developing agents. $^1H$ NMR and $^{13}C$ NMR spectra were recorded on a Bruker Avance III HD 300 MHz, a Bruker Avance III HD 400 MHz equipped with standard 5 mm probe heads or a Bruker Avance III 600 equipped with a 5 mm TCI cryoprobe. Chemical shifts were referenced to the deuterated solvent (e.g., for $CDCl_3$, δ = 7.26 ppm and 77.16 ppm for $^1H$ and $^{13}C$ NMR, respectively [1]) and reported in parts per million (ppm, δ) relative to tetramethyl silane (TMS, δ = 0.00 ppm). Coupling constants (*J*) were reported in Hz and the splitting abbreviations used were: s, singlet; d, doublet; t, triplet; m, multiplet; br, broad. Infrared spectra were recorded as FTIR spectra using a diamond ATR unit and are reported in terms of frequency of absorption (ν, $cm^{-1}$). High-resolution mass spectra were recorded on a Waters QTof-Ultima 3-Instrument with Lockspray™-Interface and a suitable external calibrant. HPLC-MS analysis were performed on a 1200 series HPLC-System with an UV diode array detector coupled with a LC/MSD trap XCT mass spectrometer (Agilent Technologies). Eluent A was acetonitrile and B was water (with 0.1% formic acid). The flow rate was 1 mL/min with a gradient 10%A to 90% A in 4 min. An Ascentis Express™ C18 column (2.7 µm, 30 mm × 2.1 mm) was used at a temperature of 40 °C. Analytical HPLC analysis were performed with an ACE C18-PFP column (125 mm × 4.6 mm, 3 µm, 40 °C) on a Knauer System with binary pump and integrated diode array detector at a flow rate of 1 mL/min. Preparative HPLC was performed with an ACE C18-PFP column (150 mm × 30 mm, 5 µm, 20 °C) and ACE C18 column (150 mm × 21.2 mm, 5 µm, 20 °C) on a Knauer System with binary pump and integrated diode array detector. For the ACE C18-PFP, a flow rate of 37.5 mL/min and for the ACE C18, a flow rate of 17.5 mL/min was used. Eluent A was acetonitrile and eluent B was water unless otherwise noted. Ozone was produced by corona discharge

method with an ozone generator from Sander. Pure oxygen (purity 4.0) was used as a supply with a flow of 100 mL/min. Solid phase peptide synthesis was performed by hand in a Merrifield reactor and with an orbital shaker. The coupling of Fmoc-D-Glu(OAll)OH, Fmoc-*L*-Tyr(O*t*-Bu)OH and Cbz-L-Thr(OH)OH was performed with 5 eq of amino acid, 4.9 eq HATU and 10 eq of NMM for 2 h in NMP. The coupling of Fmoc-D-*allo*-Thr(O*t*-Bu)OH and Fmoc-D-*allo*-IleOH was performed twice with 2 equiv of amino acid, 1.9 equiv HATU and 4 equiv of NMM for 3 h in NMP. Fmoc cleavage was achieved by treatment with 20% of piperidine in DMF (3 × 10 min). Capping was performed with a mixture of DMF, DIPEA and $Ac_2O$ (90:5:5, 2 × 10 min). Loading of the resin was determined after each coupling step by UV spectrometry of the piperidine dibenzofulvene adduct.

## *cis*-Docos-13-enylamine

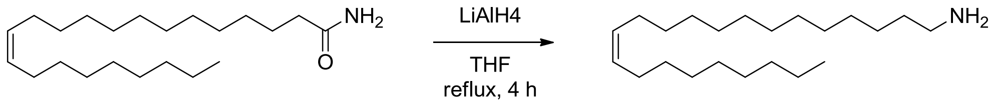


**Compound 9**

To a solution of LAH (2.30 g, 29.6 mmol) in dry THF (100 mL) was added a solution of *cis*-docosenoamide (**6**) (10.0 g, 29.7 mmol) in dry THF (100 mL) and refluxed. After 12 h the reaction mixture was cooled and $Na_2SO_4 \cdot 10H_2O$ was added slowly until the reaction ceased. The solution was filtered and concentrated in vacuo to afford *cis*-docos-13-enylamine (**9**) (9.55 g, 29.6 mmol, quant.) as a pale yellow oil.

$\mathbf{R_f}$ = 0.41 (methanol/pyridine/triethylamine 4:4:1).

**IR** (ATR) ν ($cm^{-1}$) = 3322, 3005, 2920, 2851, 1641, 1556, 1465, 1341, 721.

**$^1$H NMR** (300 MHz, $CDCl_3$) *δ* (ppm) = 5.34 (t, 2H, 13-H, 14-H), 2.67 (t, 2H, 1-H), 2.01 (q, 4H, 12-H, 15-H), 1.43 (t, 2H, 2-H), 1.26 (m, 30H), 0.88 (t, 3H, 22-H).

**$^{13}$C NMR, HSQC, HMBC** (75 MHz, $CDCl_3$) *δ* (ppm) = 130.1 (C-13, C-14), 42.4 (C-1), 33.9 – 22.9 (C-2 – C-12, C-14 – C-21), 14.3 (C-22).

## 9*H*-fluoren-9-ylmethyl (13*Z*)-docos-13-en-1-ylcarbamate

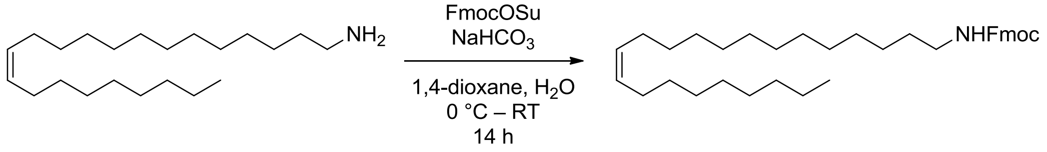

**Compound 10**

*cis*-Docos-13-enylamine (**9**) (1.00 g, 3.10 mmol) was dissolved in 1,4-dioxane (40 mL) and a saturated solution of $NaHCO_3$ in water (20 mL) was added. After cooling the reaction mixture to 0 °C, a solution of Fmoc-OSu (1.26 g, 3.72 mmol) in 1,4-dioxane (10 mL) was added and stirred for 14 h at room temperature. A saturated solution of $NaHSO_4$ in water (20 mL) was added and the mixture was extracted three times with ethyl acetate (40 mL each). After drying the combined organic phases over $Na_2SO_4$, the solvent was removed in vacuo and the residue was purified by flash column chromatography (silica, petroleum ether/$Et_2O$, gradient 0% to 20% $Et_2O$, Isolera™ Flash Purification System) to afford the title compound (1.48 g, 2.71 mmol, 87%) as a colorless solid.

**$R_f$**: 0,32 (cyclohexane/ethyl acetate 4:1).

**Mp**: 57–58 °C.

**IR** (ATR) ν ($cm^{-1}$) = 3340, 3007, 1688, 1465, 1451, 1259, 1145, 1104, 780, 757.

**$^1$H NMR, COSY** (400 MHz, $CDCl_3$) *δ* (ppm) = 7.77 (d, J = 7.4 Hz, 2H, H–4'/5'), 7.60 (d, J = 7.4 Hz, 2H, H–1'/8'), 7.40 (t, J = 7.4 Hz, 2H, H–3'/6'), 7.32 (td, J = 7.4, 1.2 Hz, 2H, H–2'/7'), 5.42 – 5.29 (m, 2H, H-13, H-14), 4.75 (m, 1H, –N*H*–), 4.41 (d, J = 6.9 Hz, 2H, H–10'), 4.22 (t, J = 6.9 Hz, 1H, H–9'), 3.19 (q, J = 6.7 Hz, 2H, H-1), 2.09 – 1.95 (m, 4H, H-12, H-15), 1.49 (m, 2H), 1.38 – 1.20 (m, 30H), 0.93 – 0.85 (m, 3H, H-22).

**$^{13}$C NMR, HSQC, HMBC** (100.6 MHz, $CDCl_3$) *δ* (ppm) = 156.5 (–NH*C*(O)O–), 144.2 (C–9a'/8a'), 141.5 (C–4a'/4b'), 130.0 (C-13/C-14), 130.0 (C-13/C-14), 127.8 (C–3'/6'), 127.1 (C–2'/7'), 125.2 (C–1'/8'), 120.1 (C–4'/5'), 66.6 (C–10'), 47.5 (C–9'), 41.3 (C–1), 32.1, 30.1, 29.9, 29.8, 29.8, 29.7, 29.7, 29.5, 29.5, 27.4 (overlapping C-12, C-15), 26.9, 22.8, 14.3 (C-22).

**ESI-HRMS**: calcd. for $[C_{37}H_{55}NO_2 + Na]^+$: 568.4131 m/z, found: 568.4122 m/z.

## 9*H*-fluoren-9-ylmethyl (13-oxotridecyl)carbamate

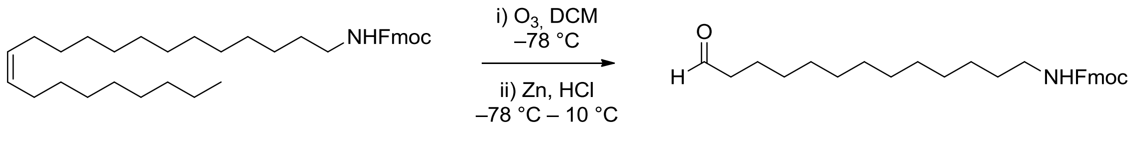


**Compound 5**

At –78 °C, ozone (made from oxygen 4.0) was bubbled through a solution of **10** (1.50 g, 2.75 mmol) in dry DCM (50 mL) until the characteristic blue color could be observed. After argon was bubbled through the solution for 30 min, zinc powder (3.50 g, 53.5 mmol) and acetic acid (4.00 mL, 70 mmol) was added and the solution was allowed to warm up to 10 °C. After 30 min, the solution was filtered, washed with water (50 mL) and a

saturated solution of $NaHCO_3$ in water (50 mL). The solvent was removed through lyophilization and the residue was purified by flash column chromatography (silica, petroleum ether/$Et_2O$, gradient 30% to 35% $Et_2O$, Isolera™ Flash Purification System) to afford the title compound (785 mg, 1.80 mmol, 66%) as a colorless solid.

**$R_f$**: 0.21 (petroleum ether/$Et_2O$ 1:1).

**Mp**: 83–84 °C.

**IR** (ATR) ν ($cm^{-1}$) = 3330, 2922, 2852, 1719, 1689, 1540, 1466, 1450, 1264, 1146, 758, 739.

**$^1$H NMR, COSY** (400 MHz, $CDCl_3$) $\delta$ (ppm) = 9.76 (t, *J* = 1.9 Hz, 1H, H–1), 7.76 (d, J = 7.4 Hz, 2H, H–4'/5'), 7.60 (d, J = 7.4 Hz, 2H, H–1'/8'), 7.40 (t, J = 7.4 Hz, 2H, H–3'/6'), 7.32 (td, J = 7.4, 1.2 Hz, 2H, H–2'/7'), 4.78 (m, 1H, –N*H*–), 4.40 (d, *J* = 7.0 Hz, 2H, H–10'), 4.22 (t, *J* = 7.0 Hz, 1H, H–9'), 3.19 (q, *J* = 6.7 Hz, 2H, H–13), 2.41 (td, *J* = 7.3, 1.9 Hz, 2H, H–2), 1.62 (p, *J* = 7.3 Hz, 2H, H–3), 1.56 – 1.44 (m, 2H, H–12), 1.35 – 1.18 (m, 17H).

**$^{13}$C NMR, HSQC, HMBC** (100.6 MHz, $CDCl_3$) $\delta$ (ppm) = 203.11 (C–1), 156.5 (–NH*C*(O)O–), 144.2 (C–9a'/8a'), 141.4 (C–4a'/4b'), 127.8 (C–3'/6'), 127.1 (C–2'/7'), 125.2 (C–1'/8'), 120.1 (C–4'/5'), 66.6 (C–10'), 47.5 (C–9'), 44.0 (C–2), 41.2 (C–13), 30.1 (C–12), 29.7, 29.5, 29.5, 29.4, 29.3, 26.9, 22.2 (C–3).

**ESI-HRMS**: calcd. for $[C_{28}H_{37}NO_3 + Na]^+$: 458.2671 m/z, found: 458.2677 m/z.

## 9*H*-fluoren-9-ylmethyl (13-hydroxyhexadec-15-en-1-yl)carbamate

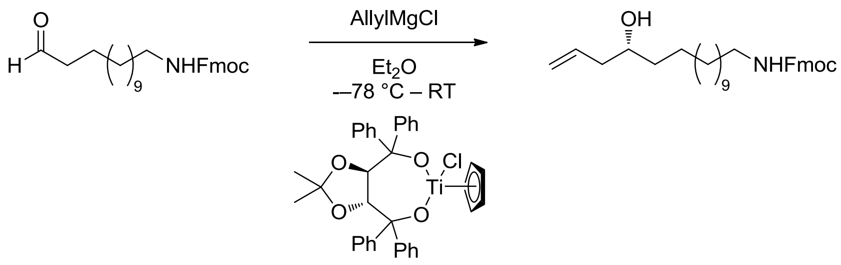


**Compound 11**

A solution of AllylMgCl (1.19 mL, 1.6 M in THF) was added at 0 °C to a solution of (*R,R*)-Duthaler–Hafner reagent (1.37 g, 2.24 mmol) in dry and degassed $Et_2O$ (30 mL). Stirring was continued for 1 h at 0 °C and then the solution was allowed to warm up to rt and stirred for additional 3 h. After cooling to –78 °C, aldehyde **10** (750 mg, 1.72 mmol) was added and the reaction was stirred at –78 °C for 4 h. Water (20 mL) was added and stirring was continued for 12 h at room temperature. A saturated aqueous solution of $NH_4Cl$ (20 mL) was added and the mixture was extracted three times with DCM (40 mL each). After drying the combined organic phases over $Na_2SO_4$, the solvent was removed

in vacuo and the residue was purified by flash column chromatography (silica, petroleum ether/$Et_2O$, gradient 30% to 40% $Et_2O$, Isolera™ Flash Purification System) to afford the title compound (690 mg, 1.45 mmol, 84%) as a colorless solid.

**$R_f$**: 0.38 (petroleum ether/$Et_2O$ 1:1).

**Mp**: 87–88 °C.

**Mosher Analysis:**

Racemate of compound **11** was synthesized by using the zinc-mediated aqueous *Barbier-Grignard* reaction [2]. Mosher ester of the racemate and the title compound was synthesized by Steglich esterification with *R*-α-methoxy-α-(trifluoromethyl)phenylacetic acid [3]. NMR analysis at 600 MHz show split signals at 5.80–5.71 and 5.67–5.59 ppm. Integration of these signals gave 94%ee.

**$[\alpha]_D^{20}$**= +2.8 (c = 1.00, $CH_2Cl_2$);

**IR** (ATR) ν ($cm^{-1}$) = 3327, 2918, 2850, 1689, 1540, 1466, 1450, 1264, 1144, 757, 737.

**$^1$H NMR, COSY** (400 MHz, $CDCl_3$) *δ* (ppm) = 7.77 (d, *J* = 7.5 Hz, 2H, H–4'/5'), 7.60 (d, *J* = 7.5 Hz, 2H, H–1'/8'), 7.40 (t, *J* = 7.5 Hz, 2H, H–3'/6'), 7.31 (td, *J* = 7.5, 1.2 Hz, 2H, H–2'/7'), 5.93 – 5.72 (m, 1H, H–15), 5.17 – 5.13 (m, 1H, H–16), 5.13 – 5.10 (m, 1H, H–16), 4.74 (s, 1H, –N*H*–), 4.40 (d, *J* = 6.9 Hz, 2H, H–10'), 4.22 (t, *J* = 6.9 Hz, 1H, H–9'), 3.64 (s, 1H, H–13), 3.19 (q, *J* = 6.7 Hz, 2H, H–1), 2.37 – 2.21 (m, 1H, H–14), 2.21 – 2.04 (m, 1H, H–14), 1.52 – 1.36 (m, 5H), 1.36 – 1.23 (m, 17H).

**$^{13}$C NMR, HSQC, HMBC** (100.6 MHz, $CDCl_3$) *δ* (ppm) = 156.5 (–NH*C*(O)O–), 144.2 (C–9a'/8a'), 141.5 (C–4a'/4b'), 135.1 (C–15), 127.8 (C–3'/6'), 127.2 (C–2'/7'), 125.2 (C–1'/8'), 120.1 (C–4'/5'), 118.2 (C–16), 70.8 (C–13), 66.6 (C–10'), 47.5 (C–9'), 42.1 (C–14), 41.3 (C–1), 37.0, 30.1, 29.8, 29.7, 29.7, 29.7, 29.4, 26.9, 25.8.

**ESI-HRMS**: calcd. for $[C_{31}H_{43}NO_3 + Na]^+$: 500.3141 m/z, found: 500.3137 m/z.

## Di-*tert*-butyl [(*E*)-{[13-(methoxymethoxy)hexadec-15-en-1-yl]amino}methylylidene]biscarbamate

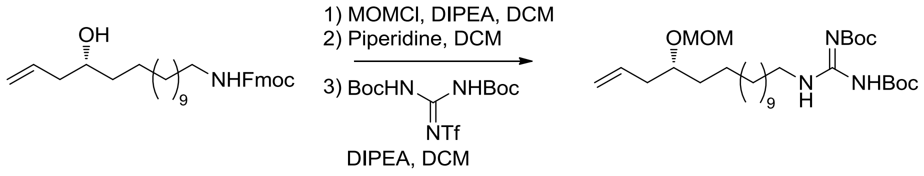


**Compound 12**

MOMCl (270 µL, 3.56 mmol, 5 equiv) was added at 0 °C to a solution of alcohol **11** (566 mg, 1.19 mmol) and DIPEA (809 µL, 4.76 mmol, 4 equiv) in dry DCM (30 mL). Stirring was continued for 1 h at 0 °C and then the solution was allowed to warm up to rt

and stirred for an additional 12 h. Water (20 mL) was added and stirring was continued for 1 h at room temperature. The mixture was extracted three times with DCM (40 mL each). After drying the combined organic layers over $Na_2SO_4$ the solvent was removed in vacuo.

The brown oil was dissolved in DCM (30 mL), piperidine (6 mL) was added and the mixture was stirred for 12 h at room temperature. The solid was removed by filtration and the filtrate was concentrated in vacuo until 20 mL of DCM were left. The mixture was filtered again and the solvent was removed completely in vacuo.

The residue was dissolved in dry DCM (10 mL) and *N*,*N′*-bis(*tert*-butoxycarboyl)-*N″*-trifylguanidine (931 mg, 2.38 mmol, 2 equiv) was added. After adding dry triethylamine (498 µL, 3.57 mmol, 3 equiv) the mixture was stirred for 5 days under nitrogen atmosphere. Solids were removed by filtration and a saturated aqueous solution of $NH_4Cl$ (20 mL) was added. The mixture was extracted three times with DCM (20 mL each) and the combined organic phases were washed with water (20 mL) and brine (20 mL). After drying the combined organic layers over $Na_2SO_4$, the solvent was removed in vacuo and the residue was purified by flash column chromatography (silica, petroleum ether/$Et_2O$, gradient 0% to 15% $Et_2O$, Isolera™ Flash Purification System) to afford the title compound (442 mg, 0.82 mmol, 49% over 3 steps) as a colorless oil.

**$R_f$**: 0.61 (petroleum ether/$Et_2O$ 7:3).

**Mp**: 87–88 °C.

**$[\alpha]_D^{20}$**= +6.4 (c = 1.00, $CH_2Cl_2$);

**IR** (ATR) ν ($cm^{-1}$) = 3336, 2926, 1719, 1639, 1616, 1415, 1367, 1332, 1155, 1133, 1043.

**$^1$H NMR, COSY** (400 MHz, $CDCl_3$) *δ* (ppm) = 11.50 (s, 1H, –N*H*Boc), 8.29 (t, *J* = 4.4 Hz, 1H, –N*H*–), 5.88 – 5.74 (m, 1H, H–15), 5.12 – 5.01 (m, 2H, H–16), 4.67 (d, *J* = 6.9 Hz, 1H, –OC$H_2$O–), 4.63 (d, *J* = 6.9 Hz, 1H, –OC$H_2$O–), 3.59 (p, *J* = 5.9 Hz, 1H, H–13), 3.43 – 3.37 (m, 2H, H–1), 3.37 (s, 3H, –OC$H_3$), 2.31 – 2.24 (m, 2H, H–14), 1.60 – 1.52 (m, 2H, H–2), 1.52 – 1.45 (m, 2H, H–12), 1.50 (s, 9H, C(C$H_3$)$_3$) 1.49 (s, 9H, C(C$H_3$)$_3$), 1.37 – 1.20 (m, 16H).

**$^{13}$C NMR, HSQC, HMBC** (100.6 MHz, $CDCl_3$) *δ* (ppm) = 163.8 (–N*C*(NBoc)NHBoc), 156.2 (–N*C*(O)O–), 153.5 (–N*C*(O)O–), 135.0 (C–15), 117.1 (C–16), 95.5 (–O*C*$H_2$O–), 83.1 (–*C*($CH_3$)$_3$), 79.3 (–*C*($CH_3$)$_3$), 77.0 (C–13), 55.6 (*–OCH$_3$*), 41.1 (C–1), 39.0 (C–14), 34.3 (C–12), 29.9, 29.8, 29.7, 29.6, 29.4, 29.1 (C–2), 28.5 (–C(*C*$H_3$)$_3$), 28.2 (–C(*C*$H_3$)$_3$), 27.0, 25.5.

**ESI-HRMS**: calcd. for $[C_{29}H_{55}N_3O_6 + Na]^+$: 564.3989 m/z, found: 564.3981 m/z.

## Di-*tert*-butyl [(*E*)-{[13-(methoxymethoxy)-15-oxopentadecyl]amino}methylylidene]biscarbamate

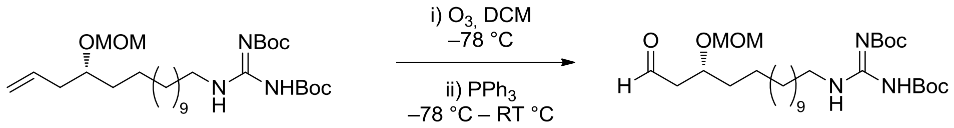


**Compound 13**

At −78 °C, ozone was bubbled through a solution of **12** (305 mg, 0.564 mmol) in dry DCM (30 mL) until the characteristic blue color could be observed. After argon was bubbled through the solution for 30 min, $PPh_3$ (0.222 g, 0.846 mmol) was added and the solution was allowed to warm up to room temperature. After 12 h the solvent was removed through lyophilization and the residue was purified by flash column chromatography (silica, petroleum ether/$Et_2O$, gradient 30% to 35% $Et_2O$, Isolera™ Flash Purification System) to afford the title compound (237 mg, 0.436 mmol, 77%) as a colorless oil.

**$R_f$**: 0.20 (petroleum ether /$Et_2O$ 7:3).

**$[\alpha]_D^{20}$**= –6.9° (c = 1.00, $CH_2Cl_2$);

**IR** (ATR) ν ($cm^{-1}$) = 3333, 2927, 1722, 1639, 1616, 1367, 1332, 1155, 1134, 1053, 1038.

**$^1$H NMR, COSY** (400 MHz, $CDCl_3$) *δ* (ppm) = 11.50 (s, 1H, –N*H*Boc), 9.80 (dd, *J* = 2.8, 1.8 Hz, 1H, H–1), 8.28 (t, *J* = 5.1 Hz, 1H, –N*H*–), 4.68 (d, *J* = 7.0 Hz, 1H, –OC$H_2$O–), 4.64 (d, *J* = 7.0 Hz, 1H, –OC$H_2$O–), 4.07 (m, 1H, H–3), 3.39 (td, *J* = 7.3, 5.1 Hz, 2H, H–15), 3.34 (s, 3H, –OCH3), 2.63 (ddd, J = 16.3, 7.0, 2.8 Hz, 1H, H–2), 2.55 (ddd, J = 16.3, 4.8, 1.8 Hz, 1H, H–2), 1.68 – 1.52 (m, 4H, H–4, H–14), 1.49 (s, 9H, C(C$H_3$)$_3$) 1.48 (s, 9H, C(C$H_3$)$_3$), 1.40 – 1.16 (m, 18H).

**$^{13}$C NMR, HSQC, HMBC** (100.6 MHz, $CDCl_3$) *δ* (ppm) = 201.6 (C–1), 163.8 (–N*C*(NBoc)NHBoc), 156.2 (–N*C*(O)O–), 153.4 (–N*C*(O)O–), 95.9 (–O*C*$H_2$O–), 83.1 (–*C*($CH_3$)$_3$), 79.3 (–*C*($CH_3$)$_3$), 73.3 (C–3), 55.8 (–O*C*$H_3$), 48.9 (C–2), 41.1 (C–15), 35.1 (C–4), 29.7, 29.7, 29.6, 29.4, 29.1, 28.5, 28.2, 27.0, 25.4.

**ESI-HRMS**: calcd. for $[C_{28}H_{54}N_3O_7 + H]^+$: 544.3962 m/z, found: 544.3938 m/z.

## 15-[*N*',*N*''-Bis(*tert*-butoxycarbonyl)carbamimidamido]-3-(methoxymethoxy)pentadecanoic acid

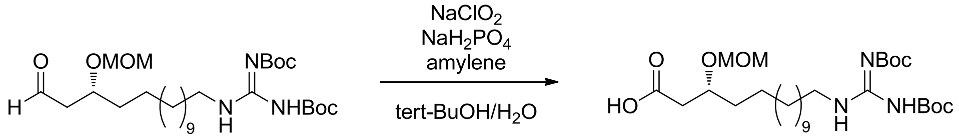

**Compound 3**

Aldehyde **13** (225 mg, 0.414 mmol) was dissolved in *tert*-butanol (5 mL) and water (5 mL). Amylene (532 µL, 4.97 mmol, 12 equiv), $NaH_2PO_4$ (521 mg, 3.73 mmol, 9 equiv) and $NaClO_2$ (227 mg, 2.48 mmol, 6 equiv) was added and the mixture was stirred for 2 h at room temperature. Water (40 mL) was added and the mixture was extracted three times with DCM (40 mL each). After drying the combined organic phases over $Na_2SO_4$ the solvent was removed in vacuo and the residue was purified by flash column chromatography (silica, cyclohexane/ethyl acetate, gradient 15% to 40% ethyl acetate, Isolera™ Flash Purification System) to afford the title compound (186 mg, 0.333 mmol, 80%) as a colorless oil.

$\mathbf{R_f}$: 0.24 (cyclohexane/ethyl acetate 7:3 + 0.1% HOAc).

$[\alpha]_D^{20}$= –4.3° (c = 1.00, $CDCl_3$);

**IR** (ATR) ν ($cm^{-1}$) = 3332, 2926, 2854, 1720, 1638, 1615, 1228, 1132, 1030, 703.

**$^1$H NMR, COSY** (400 MHz, $CDCl_3$) *δ* (ppm) = 11.47 (br s, 1H, –N*H*Boc), 8.39 (s, 1H, –N*H*–), 4.70 (d, *J* = 6.9 Hz, 1H, –OC$H_2$O–), 4.67 (d, *J* = 6.9 Hz, 1H, –OC$H_2$O–), 3.98 (p, J = 6.1 Hz, 1H, H–3), 3.46 – 3.38 (m, 2H, H–15), 3.37 (s, 3H, –OCH3), 2.68 – 2.45 (m, 2H, H–2), 1.68 – 1.52 (m, 4H, H–4, H–14), 1.50 (s, 9H, C(C$H_3$)$_3$) 1.49 (s, 9H, C(C$H_3$)$_3$),, 1.41 – 1.20 (m, 18H).

**$^{13}$C NMR, HSQC, HMBC** (100.6 MHz, $CDCl_3$) *δ* (ppm) = 176.2 (C–1), 163.8 (–N*C*(NBoc)NHBoc), 156.3 (–N*C*(O)O–), 153.5 (–N*C*(O)O–), 96.1 (–O*C*$H_2$O–), 83.2 (–*C*($CH_3$)$_3$), 79.4 (–*C*($CH_3$)$_3$), 74.7 (C–3), 55.8 (–O*C*$H_3$), 41.7 (C–15), 40.1 (C–2), 34.8 (C–4), 29.7, 29.6, 29.6, 29.6, 29.4, 29.1, 28.5, 28.2, 27.0, 25.3.

**ESI-HRMS**: calcd. for $[C_{28}H_{53}N_3O_8 + H]^+$: 560.3911 m/z, found: 560.3925 m/z.

## Cbz-L-Thr(OH)-D-*allo*-Ile-L-Tyr(OH)-D-*allo*-Thr(OH)-D-Gln-Allyl

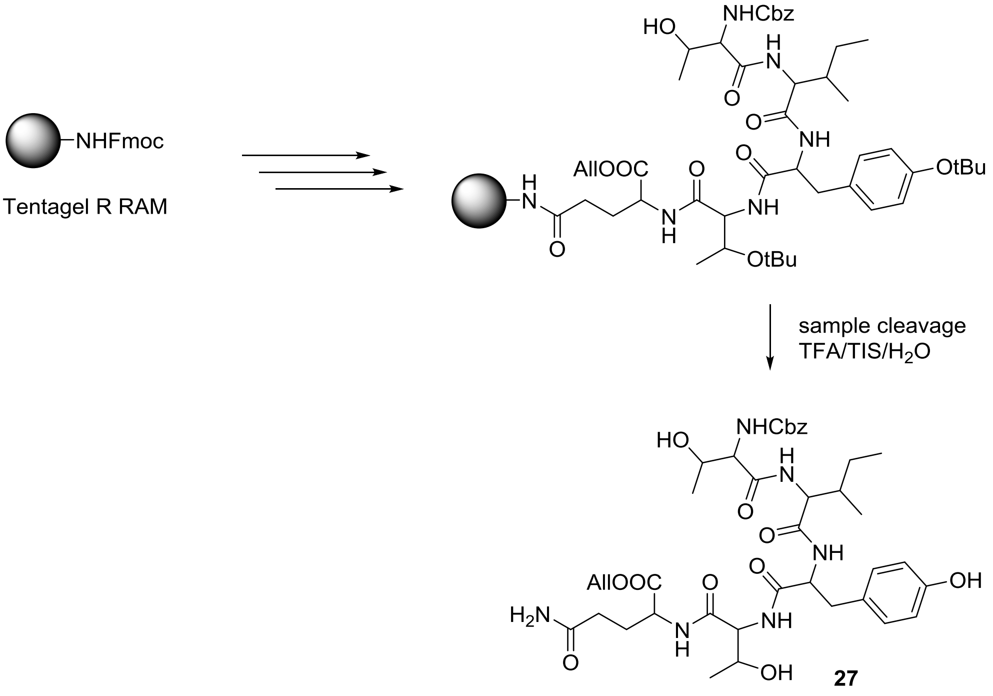


**Compound 27**

Linear peptide **27** was synthesized on a 0.6 mmol scale on a Tentagel R RAM resin using the SPPS protocol described above. Sample cleavage was done with a mixture of TFA, TIS and water (95:2.5:2.5; 30 mL/mmol) for 3 h. Triple coevaporation with toluene gave the crude peptide (5 mg) which was analyzed by HPLC–MS. Additionally, the crude peptide was purified with preparative HPLC (isocratic 30% A) to yield 4 mg of the pure Peptide which was analyzed by NMR spectroscopy.

**$^1$H NMR, COSY** (600 MHz, DMSO–d$_6$) $\delta$ (ppm) = 9.11 (s, 1H, Tyr–O*H*), 8.36 (d, *J* = 7.2 Hz, 1H, Gln–N*H*), 8.21 (d, *J* = 8.4 Hz, 1H, Tyr–N*H*), 8.12 (d, *J* = 8.8 Hz, 1H, allo-Thr–N*H*), 7.65 (d, *J* = 8.9 Hz, 1H, allo-Ile–N*H*), 7.37 – 7.28 (m, 5H, *Ph*–CH$_2$O–), 7.26 (s, 1H, Gln CO–N*H*$_2$a), 7.05 (d, *J* = 8.5 Hz, 1H, Tyr H–5), 6.98 (d, *J* = 8.7 Hz, 1H, Thr–N*H*), 6.79 (s, 1H, Gln CO–N*H*$_2$b), 6.60 (d, *J* = 8.5 Hz, 2H, Tyr H–6), 5.88 (ddt, *J* = 17.2, 10.6, 5.4 Hz, 1H, CO–CH$_2$C*H*CH$_2$), 5.31 (dq, *J* = 17.2, 1.7 Hz, 1H, CO–CH$_2$CHC*H*$_2$a), 5.20 (dq, *J* = 10.6, 1.7 Hz, 1H, CO–CH$_2$CHC*H*$_2$b), 5.03 (m, 2H, Ph–C*H*$_2$O–), 4.82 (d, *J* = 5.8 Hz, 1H, Thr–O*H*), 4.67 (d, *J* = 5.0 Hz, 1H, allo-Thr–O*H*), 4.55 (m (overlapping), 3H, Tyr H–2, CO–C*H*$_2$CHCH$_2$), 4.35 (dd, *J* = 8.8, 6.1 Hz, 1H, allo-Thr H–2), 4.30 (dd, *J* = 8.9, 4.8 Hz, 1H, allo-Ile H–2), 4.25 (ddd, *J* = 9.2, 7.2, 5.2 Hz, 1H, Gln H–2), 4.08 (m, overlapping, 2H, allo-Thr H–2, Thr H–2), 3.87 (m, overlapping, 2H, allo-Thr H–3, Thr H–3), 2.93 (dd, *J* = 13.7, 4.2 Hz, 1H, Tyr H–3a), 2.60 (m, 1H, Tyr H–3b), 2.15 (t, *J* = 7.7 Hz, 2H, Gln H–4), 1.97 (m, 1H, Gln H–3a), 1.83 (m, 1H, Gln H–3b), 1.53 (dt, *J* = 13.1, 6.8 Hz, 1H, allo-Ile H–3), 1.03 (m, 1H, allo-Ile H–4a), 1.02 (d, *J* = 6.3 Hz, 3H, Thr H–4), 0.99 (d, *J* = 6.3 Hz, 3H, allo-Thr

H–4), 0.86 (m, 1H, allo-Ile H–4b), 0.72 (t, *J* = 7.3 Hz, 3H, allo-Ile H–5), 0.47 (d, *J* = 6.8 Hz, 3H, allo-Ile H–6).

**$^{13}$C NMR, HSQC, HMBC** (151 MHz, DMSO–$d_6$) *δ* (ppm) = 173.4 (Gln C–5), 171.48 (Tyr C–1/ Gln C–1), 171.46 (Tyr C–1/ Gln C–1), 171.0 (allo-Ile C–1), 170.2 (allo-Thr C–1), 170.1 (Thr C–1), 156.1 (–N*C*(O)O–), 155.8 (Tyr C–7), 137.0 (–$Ph_{quart.}$), 132.4 (CO–$CH_2$*C*H$CH_2$), 130.2 (Tyr C–5), 128.4 (–Ph), 127.9 (Tyr C–4), 127.8 (–Ph), 127.6 (–Ph), 117.8 (CO–$CH_2$CH*C*$H_2$), 114.7 (Tyr C–6), 67.2 (allo-Thr C–3 / Thr C–3), 67.1 (allo-Thr C–3 / Thr C–3), 65.5 (Ph–*C*$H_2$O–), 64.9 (CO–*C*$H_2$CH$CH_2$), 60.1 (Thr C–2), 57.7 (allo-Thr C–2), 55.5 (allo-Ile C–2), 54.6 (Tyr C–2), 51.9 (Gln C–2), 37.3 (allo-Ile C–3), 37.2 (Tyr C–3), 31.1 (Gln C–4), 26.5 (Gln C–3), 25.8 (allo-Ile C–4), 19.7 (Thr C–4), 19.2 (allo-Thr C–4), 14.2 (allo-Ile C–6), 11.66 (allo-Ile C–5).

## Benzyl {(3*R*,6*R*,9*R*,12*S*,15*R*,18*S*,19*R*)-6-(3-amino-3-oxopropyl)-15-[(2*S*)-butan-2-yl]-12-(4-hydroxybenzyl)-9-[(1*R*)-1-hydroxyethyl]-3,19-dimethyl-2,5,8,11,14,17-hexaoxo-1-oxa-4,7,10,13,16-pentaazacyclononadecan-18-yl}carbamate

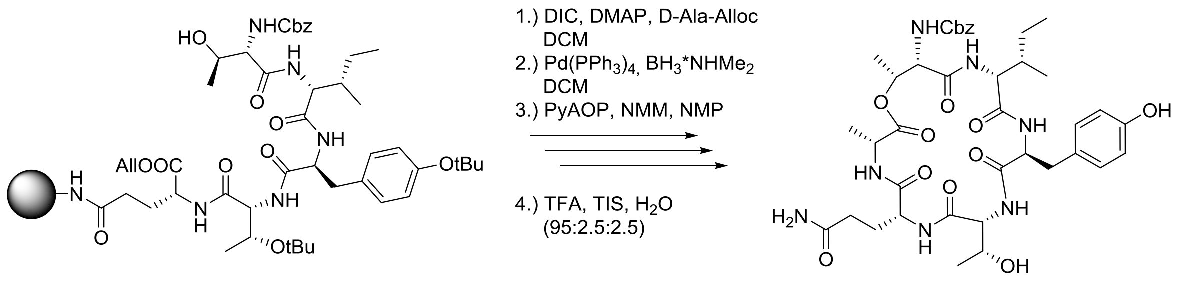


**Compound 19**

Depsipeptide **19** was synthesized by treatment of the linear peptide on a Tentagel R RAM resin in an argon atmosphere with a solution of Alloc-D-Ala-OH (2.5 eq), DIC (2.5 equiv) and DMAP (0.1 equiv) in dry DCM (3 mL/g resin) for 2 h [4]. After washing the resin with dry DCM (3 times), it was washed again with dry and degassed DCM. A solution of $BH_3$*$NHMe_2$ (20 equiv) in dry and degassed DCM (20 mL) was added and the mixture was shaken for 1 min. Then $Pd(PPh_3)_4$ (0.2 equiv) was added and the mixture was shaken for 15 min [5]. The resin was treated two more times with this mixture and washed with DCM (3 times), 0.2% TFA in DCM (3 times), DCM (3 times), 0.2% DIPEA in DCM (3 times), DCM (3 times) and NMP (3 times). Cyclization was performed 2 times with PyAOP (2 equiv) and NMM (6 equiv) in NMP (3 mL/g resin) for 2 h. After washing

with NMP (3 times) and DCM (3 times) the resin was dried and treated with a mixture of TFA, TIS and water (95:2.5:2.5; 30 mL/mmol) for 3 h. Triple coevaporation with toluene and precipitation with $Et_2O$ gave 355 mg of the crude peptide. Purification by preparative HPLC (ACE C18-PFP, 10–90% A in 20 min) yielded pure cyclodepsipeptide **19** (118 mg, 0.145 mmol, 32%).

**Mp:** 272 – 273°C

**$[\alpha]_D^{20}$**= +13.7° (c = 0.50, DMF);

**IR** (ATR) ν ($cm^{-1}$) = 3314, 2969, 1734, 1640, 1624, 1534, 1519, 1451, 1381, 1245, 1219.

**$^1$H NMR, COSY** (600 MHz, DMSO–$d_6$, mixture of diastereomeres (3:1), signals of major diastereomer) *δ* (ppm) = 9.13 (s, 1H, Tyr–O*H*), 8.58 (d, *J* = 8.4 Hz, 1H, Tyr–N*H*), 8.34 (d, *J* = 7.7 Hz, 1H, allo-Thr–N*H*), 8.04 (br s, Gln–N*H*), 7.59–7.46 (m, 2H, Thr–N*H*, allo-Ile–N*H*), 7.30 (m overlapping, 6H, Ala–N*H*, *Ph*–), 7.19 (br s, 1H, Gln CO–N$H_2$a), 7.04 (d, *J* = 8.3 Hz, 1H, Tyr H–5), 6.77 (br s, 1H, Gln CO–N$H_2$b), 6.60 (d, *J* = 8.3 Hz, 2H, Tyr H–6), 5.25 (m, 1H, Ala H–3), 5.10 (m, 2H, Ph–C$H_2$O–), 4.93 (br d, *J* = 5.6 Hz, 1H, allo-Thr–O*H*), 4.55 (m, 1H, Tyr H–2), 4.08 (m, 1H, Thr H–2), 4.26 (m, 1H, Ala H–2), 4.09 – 3.86 (m, 4H, Gln H–2, allo-Thr H–2, allo-Thr H–3, allo-Ile H–2), 3.02 – 2.95 (m, 1H, Tyr H–3a), 2.59 – 2.52 (m, 1H, Tyr H–3b), 2.17 – 2.00 (m, 3H, Gln H–4a, Gln H–4b, Gln H–3a), 1.98 – 1.86 (m, 1H, Gln H–3b), 1.36 – 1.29 (m, 1H, allo-Ile H–3), 1.20 – 1.07 (m, 6H, Ala H–3, Thr H–4), 1.04 – 0.93 (m, 3H, allo-Thr H–4), 0.76 – 0.66 (m, 1H, allo-Ile H–4a), 0.66 – 0.54 (m, 7H, allo-Ile H–4b, allo-Ile H–5, allo-Ile H–6)

**$^{13}$C NMR, HSQC, HMBC** (151 MHz, DMSO–$d_6$) *δ* (ppm) = 173.9 (Gln C–5), 171.8 (Tyr C–1), 170.5, 170.5, 170.4, 170.1 (Ala C–1), 169.0 (Thr C–1), 156.4 (–NH*C*(O)O–), 155.7 (Tyr C–7), 136.8 (*Ph*-quart.), 130.2 (Tyr C–5), 128.4 (*Ph*), 128.0 (Tyr C–4), 128.0 (*Ph*), 127.8 (*Ph*), 114.7 (Tyr C–6), 70.3 (Thr C–3), 66.1 (Ph–$CH_2$–), 65.6 (allo-Thr C–3), 59.6 (allo-Thr C–2), 58.4 (Thr C–2), 57.3 (allo-Ile C–2), 54.5 (Tyr C–2), 53.4 (Gln C–2), 47.9 (Ala C–2), 36.8 (all-Ile C–3), 36.7 (Tyr C–3), 31.7 (Gln C–4), 26.5 (Gln C–3), 25.0 (allo-Ile C–4), 20.2 (allo-Thr C–4), 18.1 (Ala C–3), 16.7 (allo-Thr C–4), 14.5 (allo-Ile C–5), 11.3 (allo-Ile C–6)

**ESI-HRMS**: calcd. for $[C_{39}H_{53}N_7O_{12} + Na]^+$: 834.3650 m/z, found: 834.3645 m/z.

**Di-*tert*-butyl [(*E*)-{[(13*R*)-15-({(3*R*,6*R*,9*R*,12*S*,15*R*,18*S*,19*R*)-6-(3-amino-3-oxopropyl)-15-[(2*S*)-butan-2-yl]-12-(4-hydroxybenzyl)-9-[(1*R*)-1-hydroxyethyl]-3,19-dimethyl-2,5,8,11,14,17-hexaoxo-1-oxa-4,7,10,13,16-pentaazacyclononadecan-18-yl}amino)-13-(methoxymethoxy)-15-oxopentadecyl]amino}methylylidene]biscarbamate**

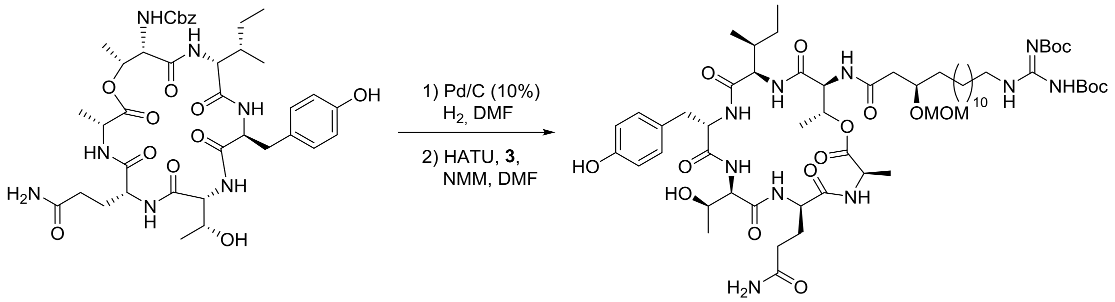


**Compound 24**

Cyclodepsipeptide **19** (19 mg, 23.4 µmol) was dissolved in DMF (8 mL) and palladium on charcoal (2 mg, 10 wt % Pd on carbon) was added. After stirring for 12 h under hydrogen atmosphere (1 bar), the mixture was filtered and the solvent was removed in vacuo to yield the amine (16 mg).

Protected GHPD acid **3** (21.7 mg, 38.8 µmol, 2 equiv) was dissolved in DMF (2 mL), HATU (13.9 mg, 36.6 mmol, 1.9 equiv) and NMM (16.0 µL, µmol, 7.6 equiv) were added and the mixture was stirred for 5 min. After this time span, amine (13.0 mg, 19.2 µmol) was added and the mixture was stirred for 70 h at room temperature. Removal of the solvent in vacuo and purification by preparative HPLC (ACE C18 60% A for 4 min, then gradient to 90% A in 10 min) yielded pure cyclodepsipeptide **24** (3.8 mg, 3.12 µmol, 16%).

**ESI-HRMS**: calcd. for $[C_{59}H_{98}N_{10}O_{17} + Na]^+$: 1241.7009 m/z, found: 1241.6992 m/z.

**$^1$H NMR, COSY** (600 MHz, DMSO–$d_6$) *δ* (ppm) = 11.49 (s, 1H, –N*H*Boc), 9.09 (s, 1H, Tyr–OH), 8.50 (d, *J* = 8.5 Hz, 1H, Tyr–N*H*), 8.45 (d, *J* = 5.5 Hz, 1H, allo-Thr–N*H*), 8.34 (br s, 1H, Gln–N*H*), 8.28 (t, *J* = 5.6 Hz, 1H, –N*H*C(NBoc)NHBoc), 7.86 (d, *J* = 8.4 Hz, 1H, Thr–N*H*), 7.20 – 7.15 (m, 2H, allo-Ile–N*H*, Gln–N$H_2$a), 7.06 (d, *J* = 8.5 Hz, 3H, Ala–N*H*, Tyr H–5), 6.77 (br s, 1H, Gln–N$H_2$b), 6.59 (d, J = 8.5 Hz, 2H, Tyr H–6), 5.39 – 5.33 (m, 1H, Thr H–3), 5.03 (br s, 1H, allo-Thr–O*H*), 4.67 (d, *J* = 6.8 Hz, 1H, –OC$H_2$O–), 4.64 – 4.56 (m, 2H, –OC$H_2$O–, Tyr H–2), 4.40 – 4.29 (m, 2H, Ala H–2, Thr H–2), 4.19 (t, *J* = 8.8 Hz, 1H, allo-Ile H–2), 3.85 (m, 3H, allo-Thr H–2, allo-Thr H–3, GHPD H–3), 3.73 (br s, 1H, Gln H–2), 3.29 – 3.24 (m, 2H, GHPD H–15), 3.24 (s, 3H, –OC$H_3$), 2.86 (dd, *J* = 13.7, 3.7 Hz, 1H, Tyr H–3a), 2.64 – 2.57 (m, 2H, Tyr H–3b, GHPD H–2a), 2.42 (dd, *J* = 13.9, 5.4 Hz, 1H, GHPD H–2b), 2.19 – 2.13 (m, 1H, Gln H–3a), 2.13 – 2.04 (m, 2H, Gln H–3b,

Gln H–4a), 2.01 – 1.93 (m, 1H, Gln H–4b), 1.53 – 1.41 (m, 11H, –C(C*H*$_3$)$_3$, GHPD H–4), 1.38 (s, 9H, –C(C*H*$_3$)$_3$), 1.31 (m, 1H, allo-Ile H–3), 1.27 – 1.20 (m, 20H, GHPD H–(5–14)), 1.15 (d, $J$ = 6.5 Hz, 3H, Thr H–4), 1.12 – 1.07 (m, 6H, Ala H–3, allo-Thr H–4), 0.59 – 0.54 (m, 4H, allo-Ile H–4a, allo-Ile H–5), 0.51 (d, $J$ = 6.7 Hz, 4H, allo-Ile H–4b, allo-Ile H–6).

**$^{13}$C NMR, HSQC, HMBC** (151 MHz, DMSO–d$_6$) $\delta$ (ppm) = 174.3 (Gln C–5), 172.5 (Tyr C–1), 171.1 (GHPD C–1), 170.9, 170.4 (Ala C–1), 170.3 , 170.2 (allo-Ile C–1), 168.3 (Thr C–1), 163.2 (–NH*C*(NBoc)NHBoc), 155.8 (Tyr C–7), 155.2 (–NH*C*(O)O–), 152.1 (–NH*C*(O)O–), 130.3 (Tyr C–5), 127.8 (Tyr C–4), 114.6 (Tyr C–6), 94.4 (–O*C*H$_2$O–), 82.9 (–*C*(CH$_3$)$_3$), 78.1 (–*C*(CH$_3$)$_3$), 73.7 (GHPD C–3), 69.8 (Thr C–3), 65.7 (allo-Thr C–3), 60.2 (allo-Thr C–2), 57.1 (Thr C–2), 56.6 (allo-Ile C–2), 54.9 (–O*C*H$_3$), 54.1 (Tyr C–2), 53.2 (Gln C–2), 47.3 (Ala C–2), 40.2 (GHPD C–15), 39.8 (GHPD C–2), 37.7 (Tyr C–3), 37.3 (allo-Ile C–3), 33.8 (GHPD C–4), 31.8 (Gln C–4), 29.1, 29.1, 29.0, 29.0, 28.6, 28.5, 28.0, 27.6, 26.2, 25.2 (Gln C–3), 24.9 (allo Ile C–4), 20.6 (allo-Thr C–4), 18.2 (Ala C–3), 16.9 (Thr C–4), 14.3 (allo Ile C–6), 11.4 (allo Ile C–5).

## (3*R*)-*N*-{(3*R*,6*R*,9*R*,12*S*,15*R*,18*S*,19*R*)-6-(3-amino-3-oxopropyl)-15-[(2*S*)-butan-2-yl]-12-(4-hydroxybenzyl)-9-[(1*R*)-1-hydroxyethyl]-3,19-dimethyl-2,5,8,11,14,17-hexaoxo-1-oxa-4,7,10,13,16-pentaazacyclononadecan-18-yl}-15-carbamimidamido-3-hydroxypentadecanamide

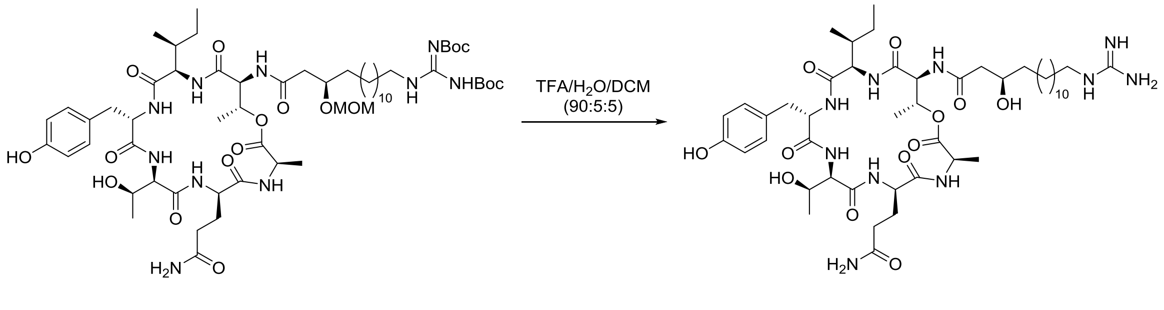


**Compound 1**

Cyclodepsipeptide **24** (3.8 mg, 3.12 µmol) was dissolved in a mixture of TFA (1.8 mL), water (0.1 mL) and DCM (0.1 mL) and stirred for 1 h at room temperature. Toluene was added and the solvent was removed in vacuo. Purification by preparative HPLC (ACE C18*,* 10% to 50% A (MeCN + 0.1% formic acid) in 15 min yielded pure cyclodepsipeptide **1** (1.8 mg, 1.85 µmol, 59%).

**$[\alpha]_D^{24}$**= +2.0° (c = 0.1, DMSO-d$_6$).

**ESI-MS/MS:** 975.6 (M$^+$), 957.6 (M–H$_2$O), 720.5 (M–GHPD)

CID [720.5]: 631.3 [M–GHPD–H$_2$O–Ala]

503.2 [M–GHPD–H$_2$O–Ala–Gln]

402.2 [M–GHPD–$H_2O$–Ala–Gln–Thr]

239.1 [M–GHPD–$H_2O$–Ala–Gln–Thr–Tyr]

All fragments are identical to those of the natural product.

**ESI-HRMS**: calcd. for [$C_{47}H_{78}N_{10}O_{12}$ + H]$^+$: 975.5879 m/z, found: 975.5854 m/z.

**$^1$H NMR, COSY** (600 MHz, DMSO–$d_6$) $\delta$ (ppm) = 9.23 (br s, 1H, Tyr–OH), 8.52 (d, $J$ = 7.8 Hz, 1H, Tyr–N$H$), 8.49 (br s, 1H, allo-Thr–N$H$), 8.20 (br s, 1H, Gln–N$H$), 7.76 (br s, 1H, Thr–N$H$), 7.64 – 7.42 (m, 1H, Ala–NH), 7.39 (br s, 1H, allo-Ile–NH), 7.26 (br s, 1H, Gln–N$H_2$a), 7.06 (d, $J$ = 8.5 Hz, 2H, Tyr H–5), 6.82 (br s, 1H, Gln–N$H_2$b), 6.60 (d, J = 8.5 Hz, 2H, Tyr H–6), 5.34 – 5.28 (m, 1H, Thr H–3), 4.90 (br s, 1H, GHPD–OH), 4.54 – 4.48 (m,1H, Tyr H–2), 4.46 (br d, $J$ = 7.8 Hz, 1H, Thr H–2), 4.24 – 4.12 (m, 2H, Ala H–2, allo-Ile H–2), 3.90 – 3.83 (m, 1H, Gln H–2), 3.94 (m, 2H, allo-Thr H–2, allo-Thr H–3), 3.80 – 3.73 (m, 1H, GHPD H–3), 3.03 (q, $J$ = 6.5 Hz,, 2H, GHPD H–15), 2.89 (d, $J$ = 12.7 Hz, 1H, Tyr H–3a), 2.59 (m, 1H, Tyr H–3b), 2.33 – 2.39 (m, 1H, GHPD H–2), 2.21 – 2.14 (m, 1H, Gln H–4a), 2.12 – 2.03 (m, 2H, Gln H–3b, Gln H–4a), 2.02 – 1.93 (m, 1H, Gln H–3a), 1.46 – 1.40 (m, 2H, GHPD H–14), 1.35 (m, 4H, allo-Ile H–3, GHPD H–4, GHPD H–5a), 1.30 – 1.19 (m, 18H, GHPD H–(5–13), GHPD H–5b), 1.17 (d, $J$ = 7.1 Hz, 3H, Ala H–3), 1.14 (d, $J$ = 6.5 Hz, 3H, Thr H–4), 1.05 (br s, 3H, allo-Thr H–4), 0.75 – 0.67 (m, 1H, allo-Ile H–4a), 0.63 – 0.59 (m, 4H, allo-Ile H–4b, allo-Ile H–5), 0.53 (d, $J$ = 6.5 Hz, 3H, allo-Ile H–6).

**$^{13}$C NMR, HSQC, HMBC** (151 MHz, DMSO–$d_6$) $\delta$ (ppm) = 174.3 (Gln C–5), 166.8 (Tyr C–1), 171.9 (GHPD C–1), 170.6 (allo-Thr C–1), 170.4 (Ala C–1), 170.4 (Gln C–1), 170.4 (allo-Ile C–1), 168.5 (Thr C–1), 157.1 (–NH$C$(NH)$NH_2$), 155.9 (Tyr C–7), 130.2 (Tyr C–5), 127.7 (Tyr C–4), 114.7 (Tyr C–6), 70.2 (Thr C–3), 67.5 (GHPD C–3), 65.6 (allo-Thr C–3), 59.9 (allo-Thr C–2), 56.5 (Thr C–2), 56.5 (allo-Ile C–2), 54.5 (Tyr C–2), 53.2 (Gln C–2), 47.8 (Ala C–2), 43.3 (GHPD C–2), 40.6 (GHPD C–15), 37.2 (allo-Ile C–3), 36.9 (Tyr C–3), 36.9 (GHPD C–4), 32.0 (Gln C–4), 29.1, 29.1, 29.0, 28.7, 28.5 (GHPD C–14), 28.0, 27.6, 26.2, 26.1 (Gln C–3), 25.4 (GHPD C–5), 25.1 (allo Ile C–4), 20.2 (allo-Thr C–4), 17.8 (Ala C–3), 16.7 (Thr C–4), 14.4 (allo Ile C–6), 11.4 (allo Ile C–5).

## III) ¹H and ¹³C NMR spectra of compounds

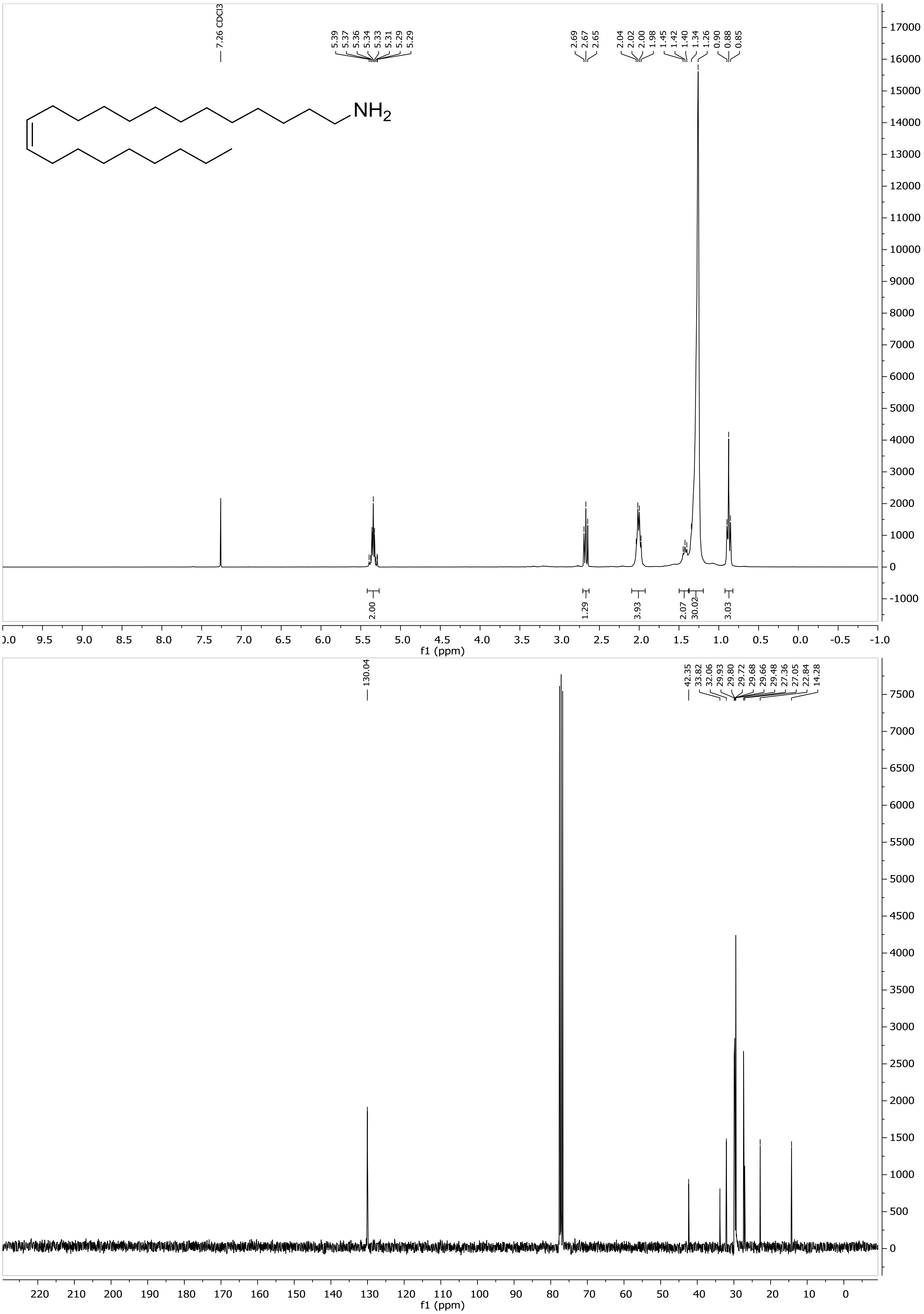


**Figure S1: $^{1}$H-NMR (300 MHz, $CDCl_3$) and $^{13}$C-NMR (75 MHz, $CDCl_3$) of compound 9**

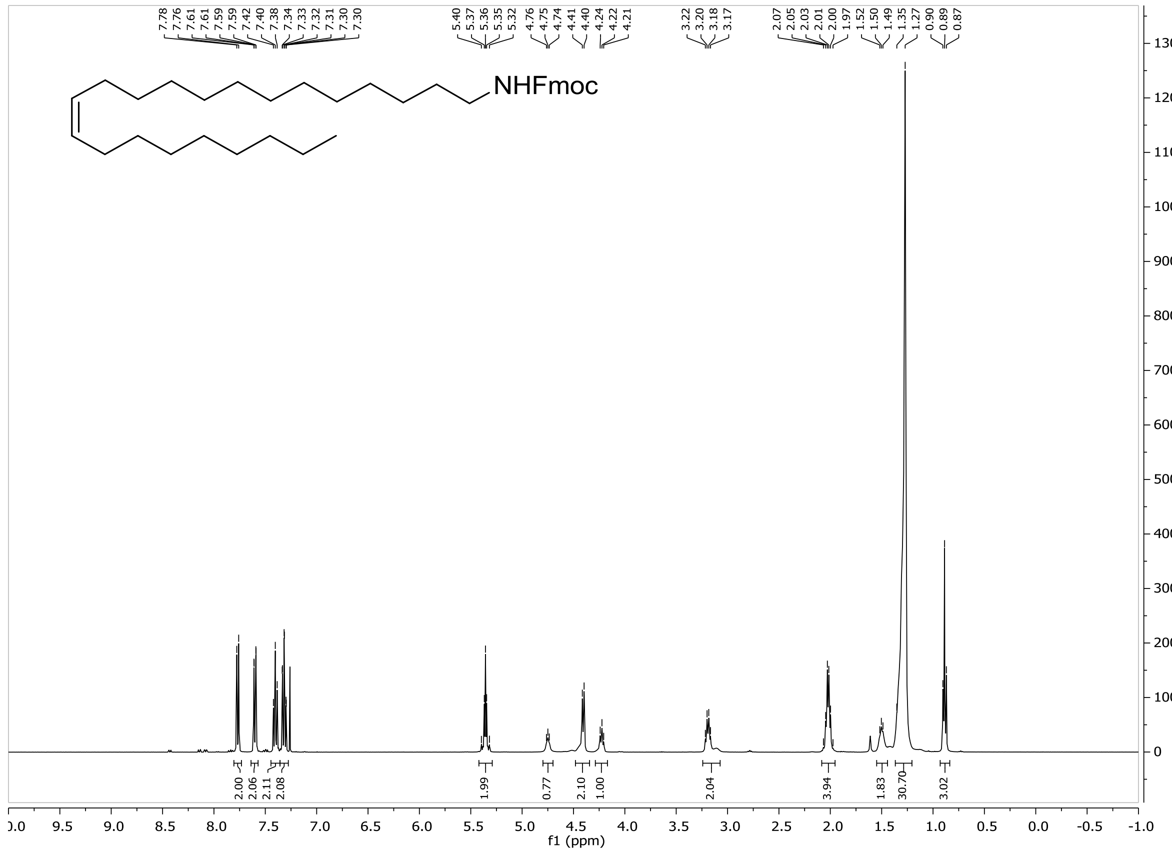


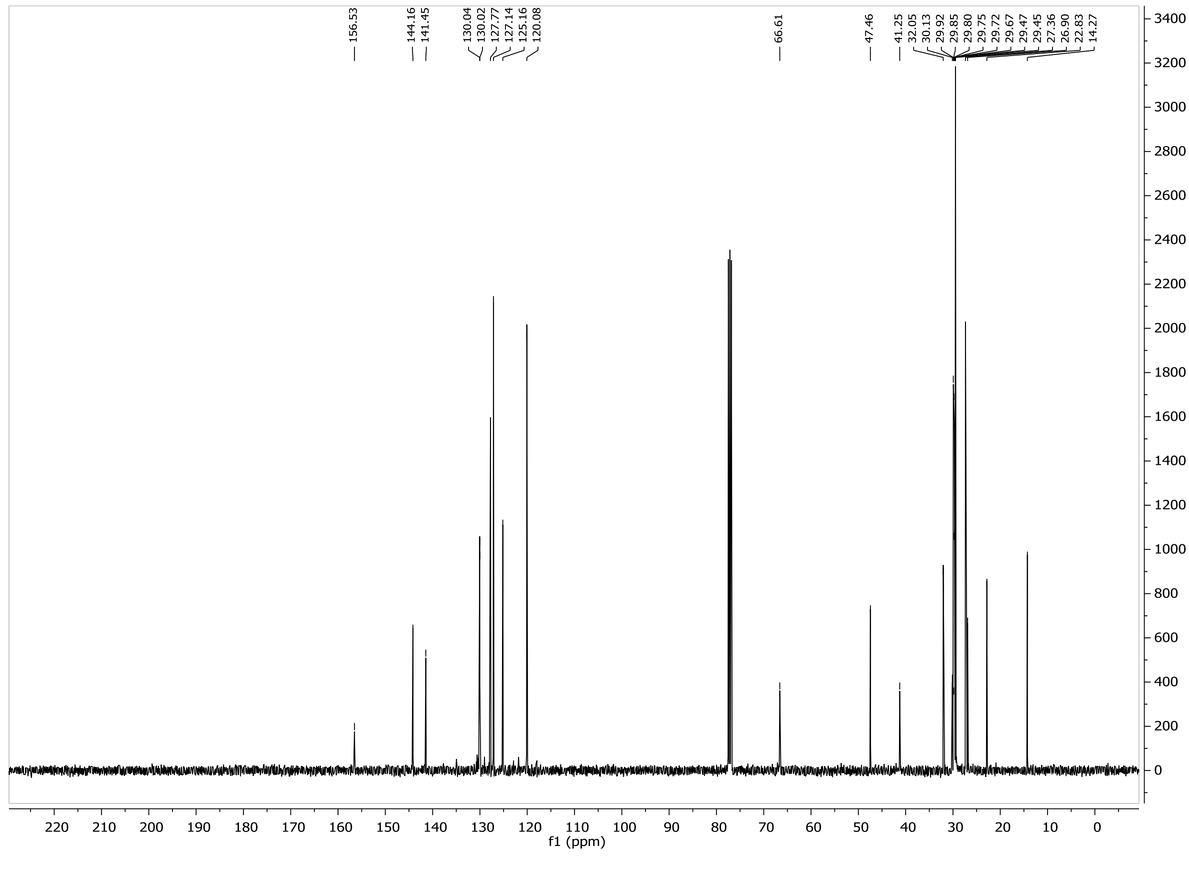


**Figure S2: $^{1}$H-NMR (400 MHz, $CDCl_3$) and $^{13}$C-NMR (101 MHz, $CDCl_3$) of compound 10**

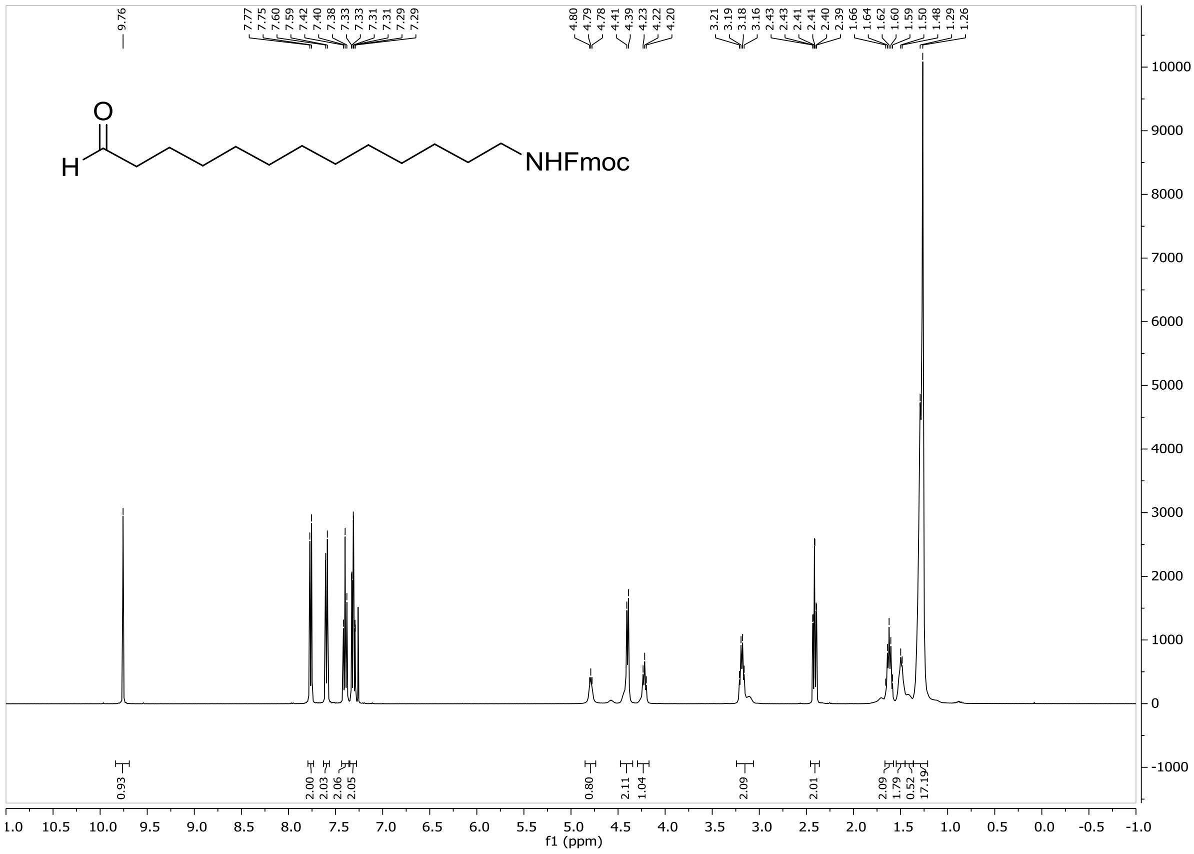

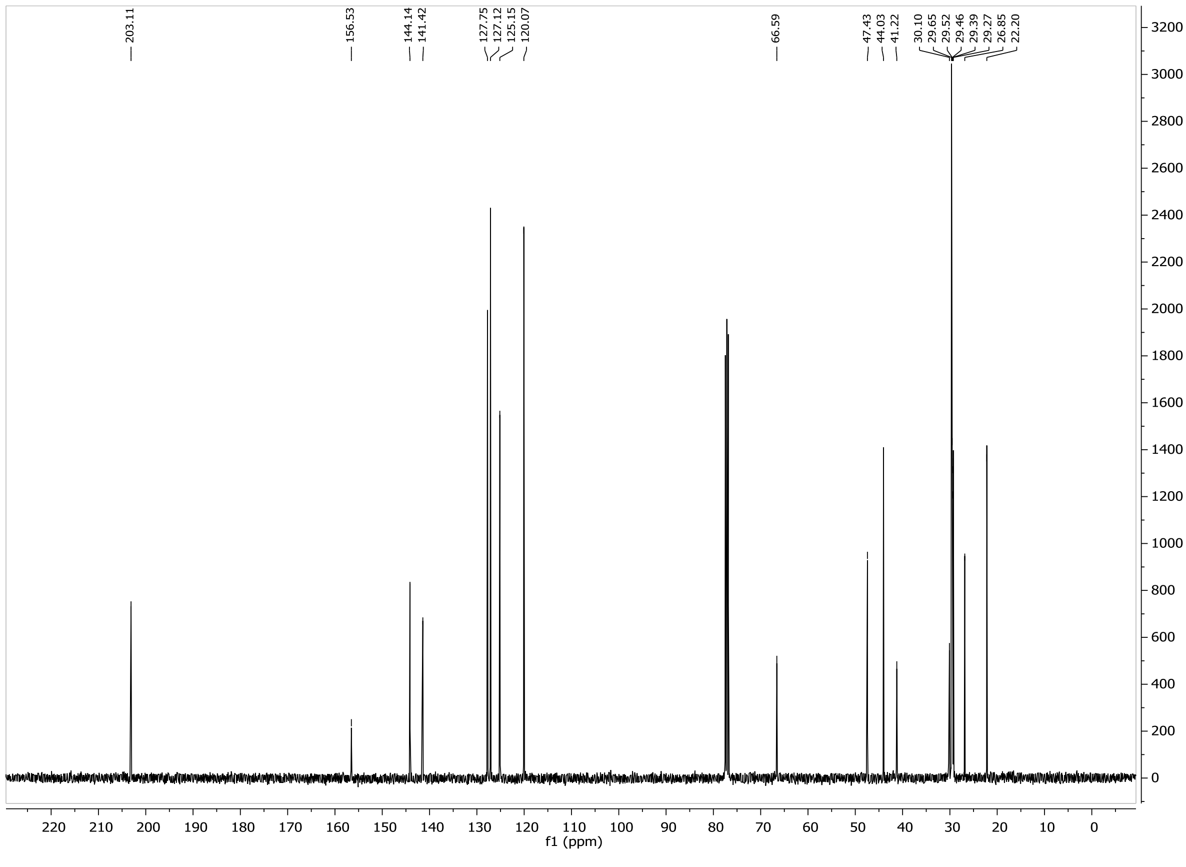

**Figure S3: $^1$H-NMR (400 MHz, $CDCl_3$) and $^{13}$C-NMR (101 MHz, $CDCl_3$) of compound 5**

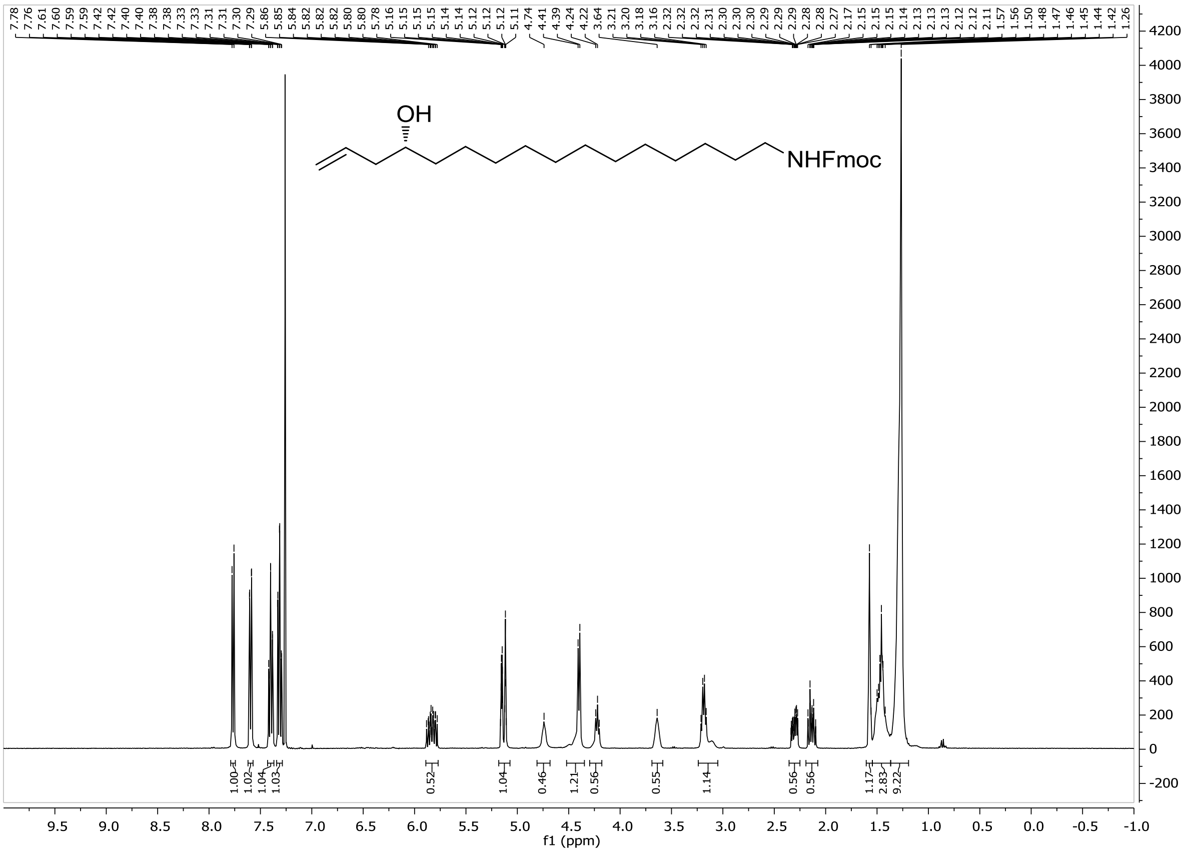


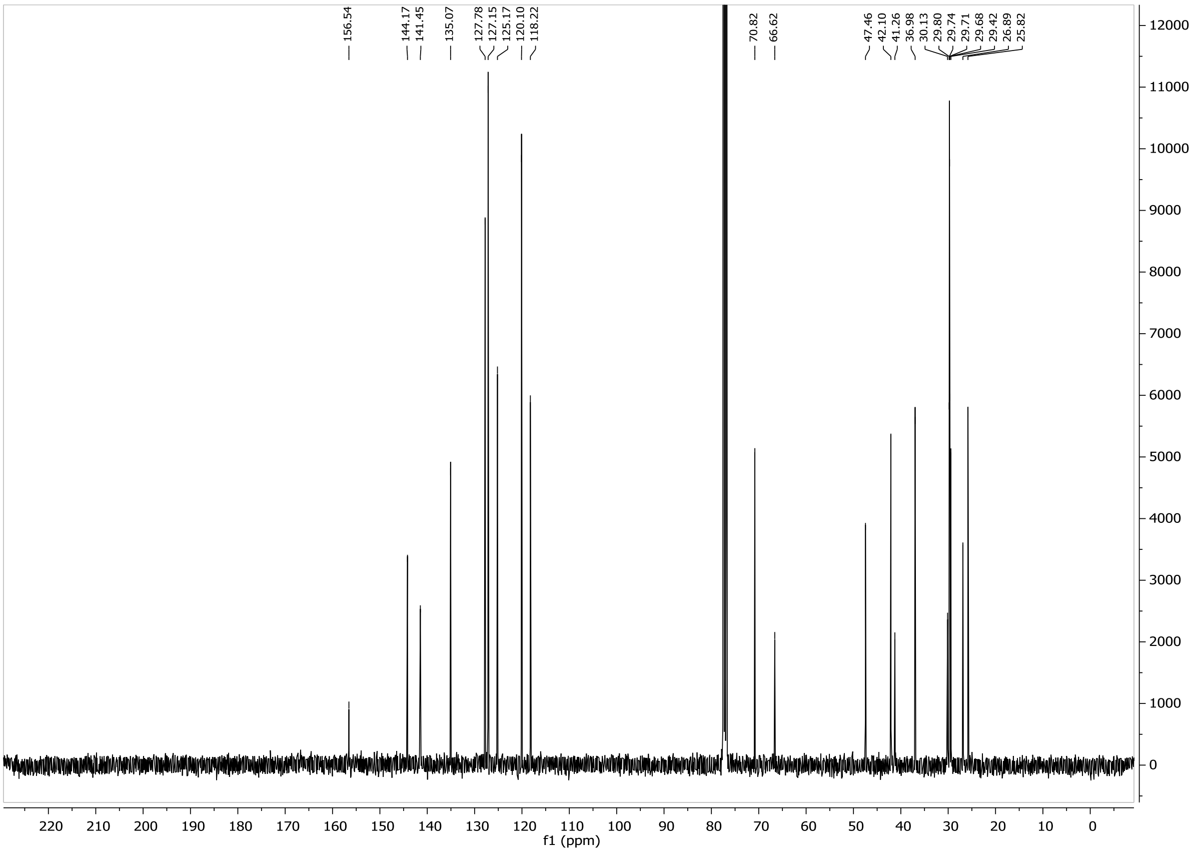


**Figure S4: $^{1}$H-NMR (400 MHz, $CDCl_3$) and $^{13}$C-NMR (101 MHz, $CDCl_3$) of compound 11**

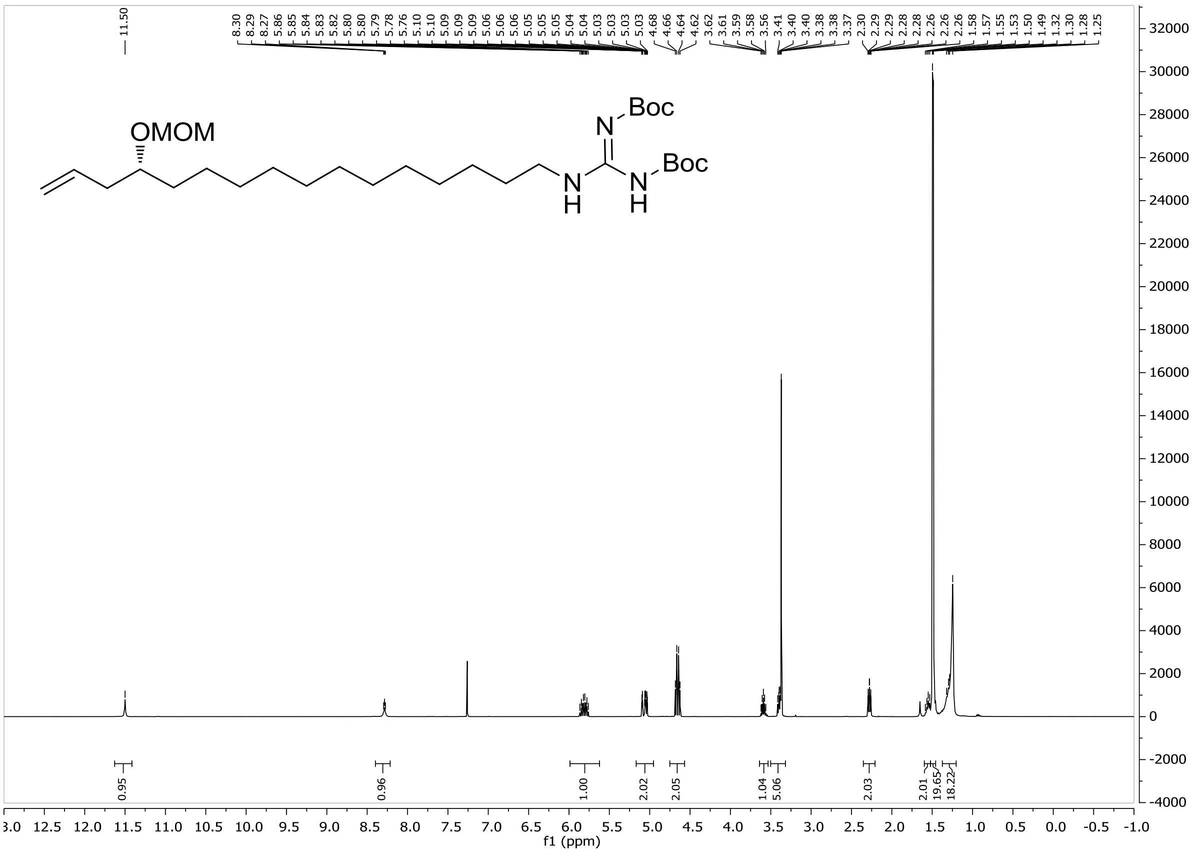


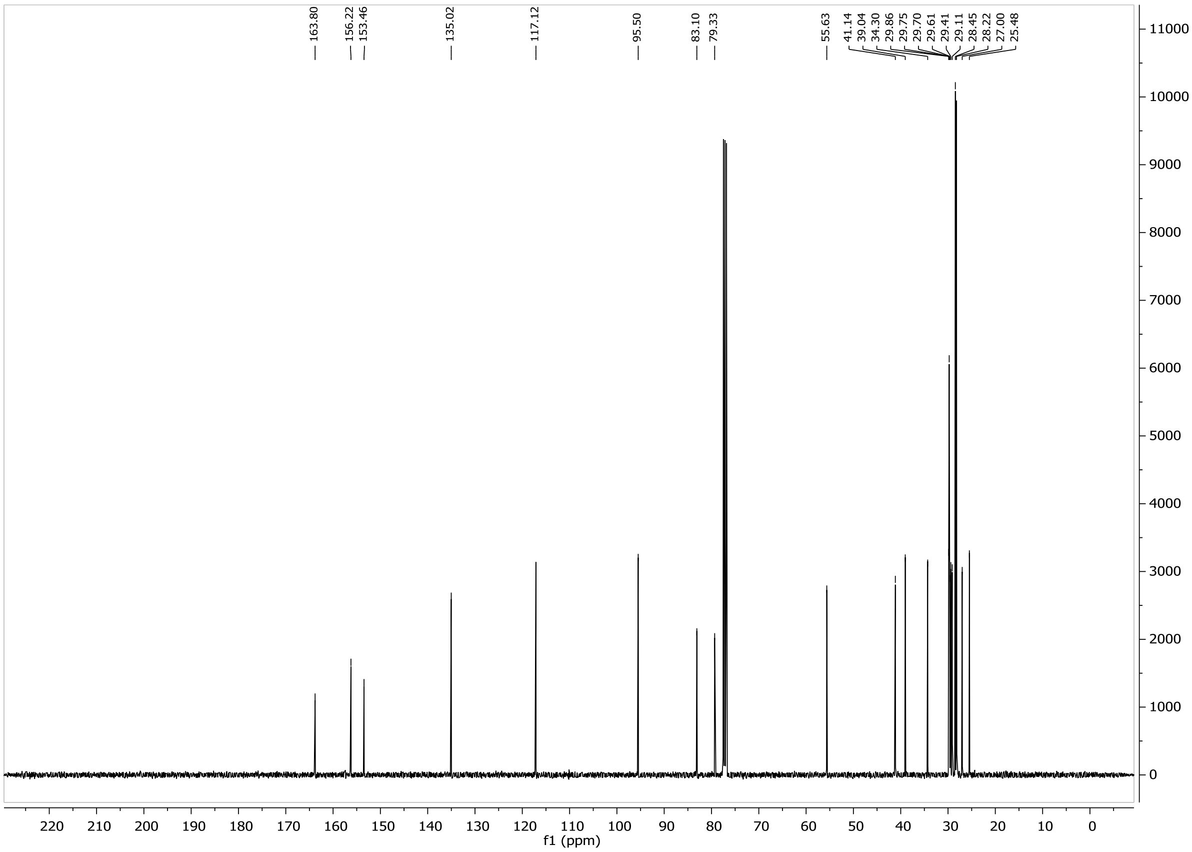


**Figure S5: $^{1}$H-NMR (400 MHz, $CDCl_3$) and $^{13}$C-NMR (101 MHz, $CDCl_3$) of compound 12**

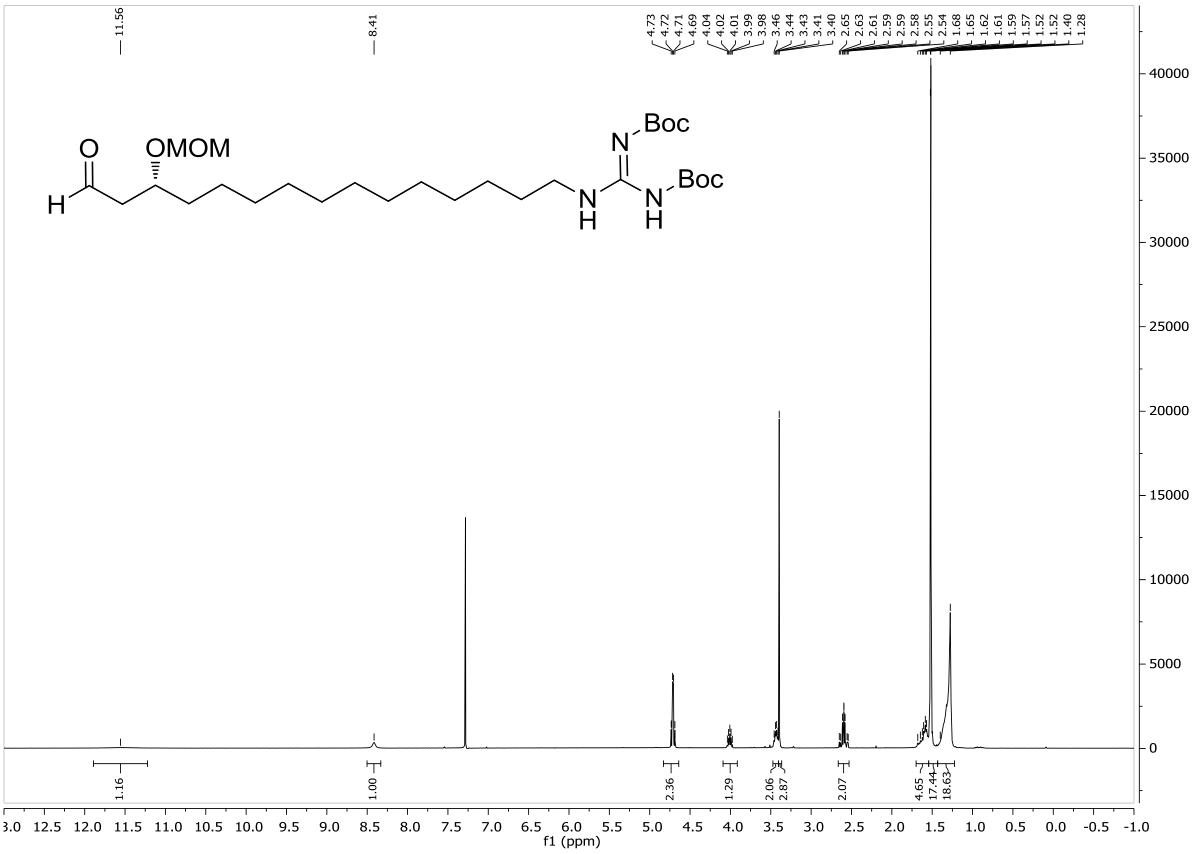


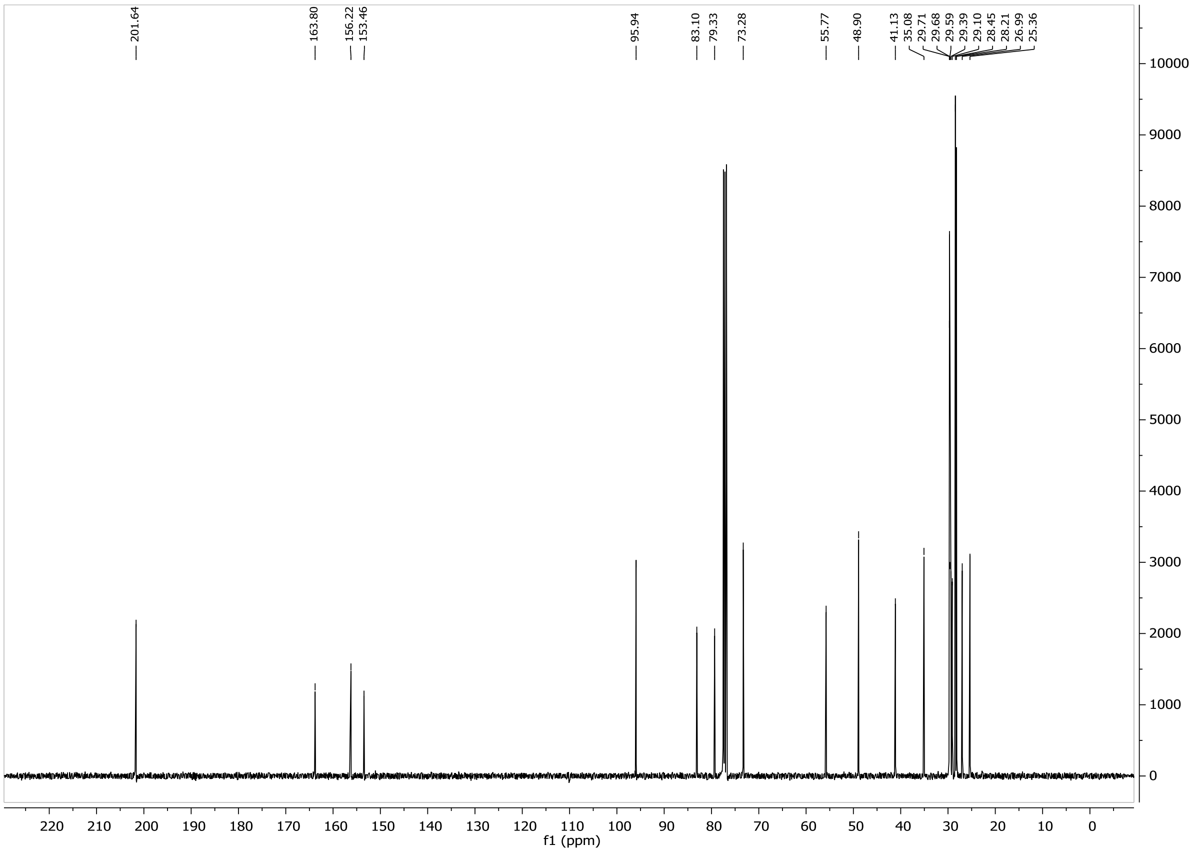


**Figure S6: $^1$H-NMR (400 MHz, $CDCl_3$) and $^{13}$C-NMR (101 MHz, $CDCl_3$) of compound 13**

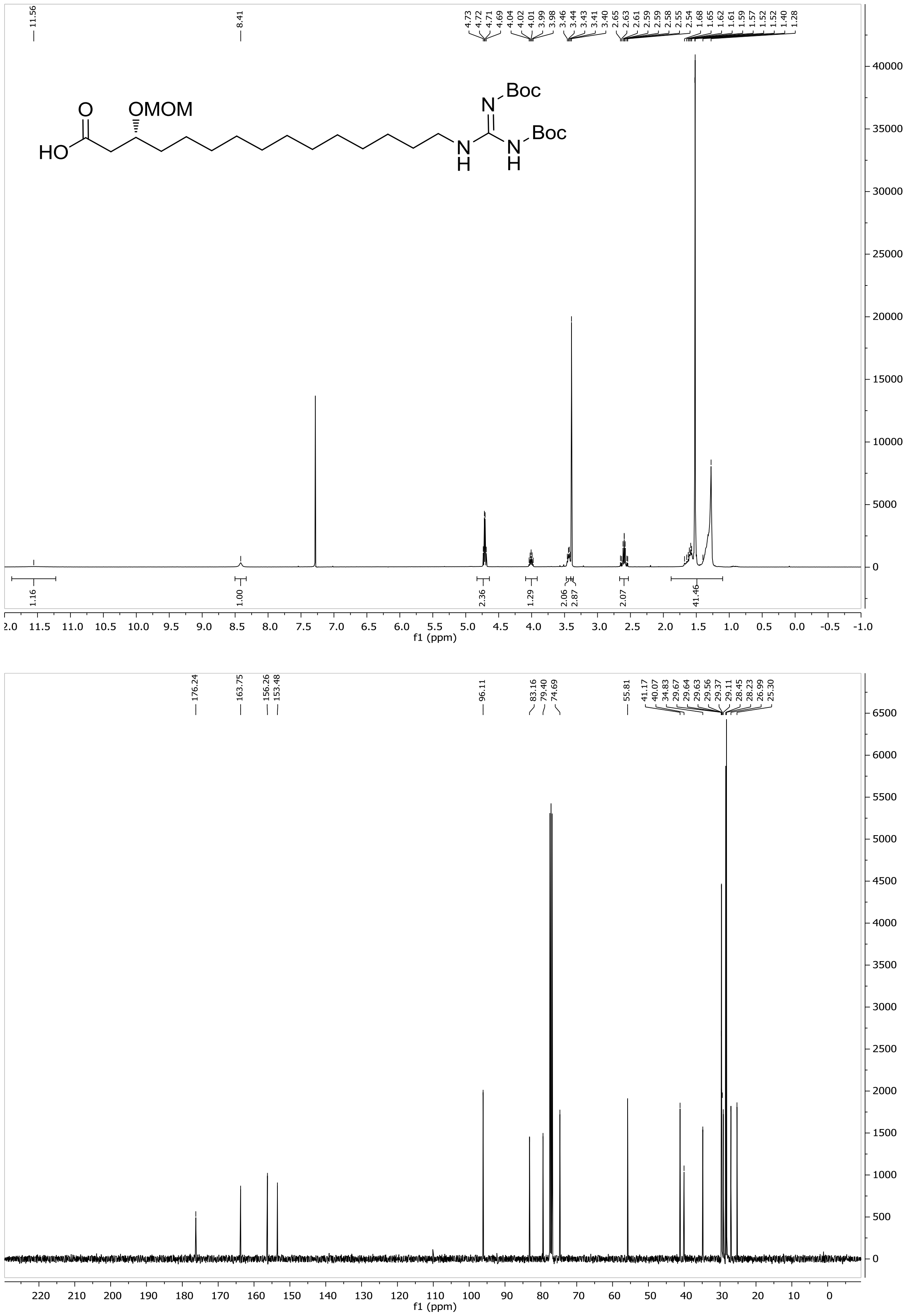


**Figure S7: $^{1}$H-NMR (400 MHz, $CDCl_3$) and $^{13}$C-NMR (101 MHz, $CDCl_3$) of compound 3**

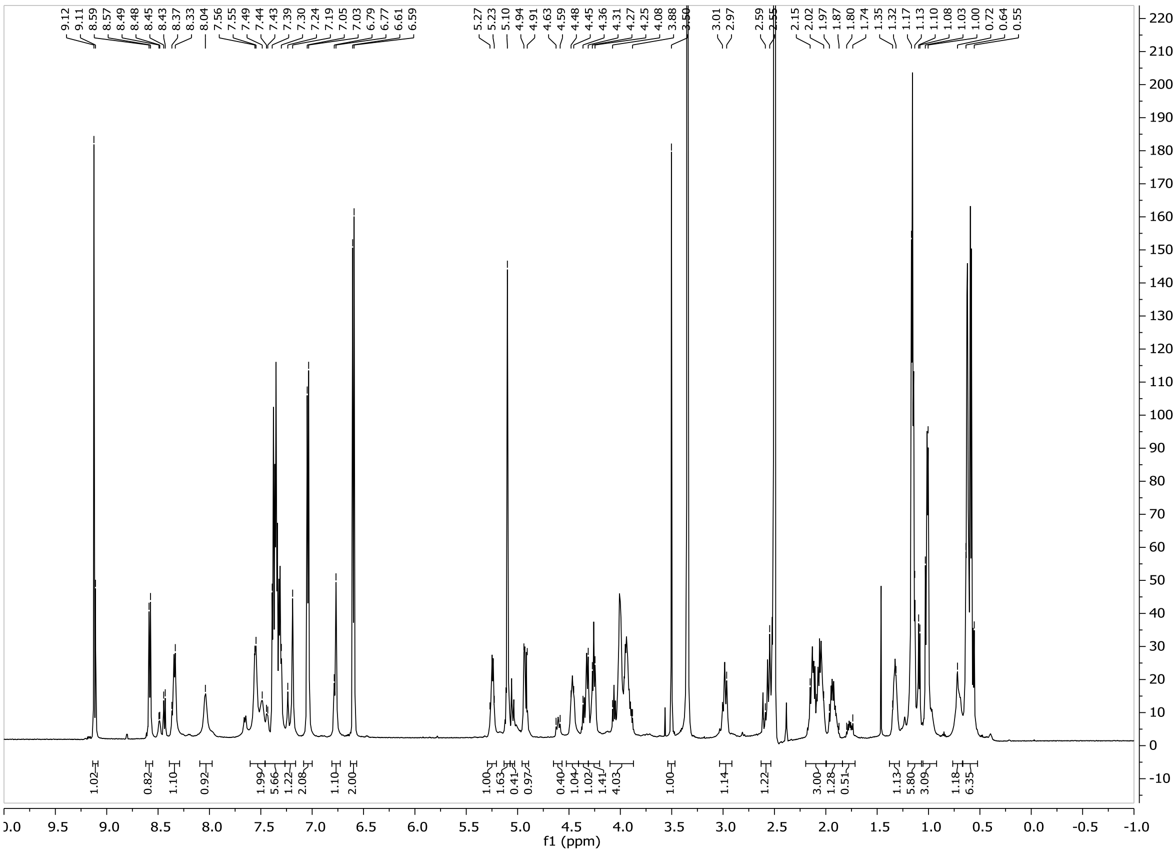

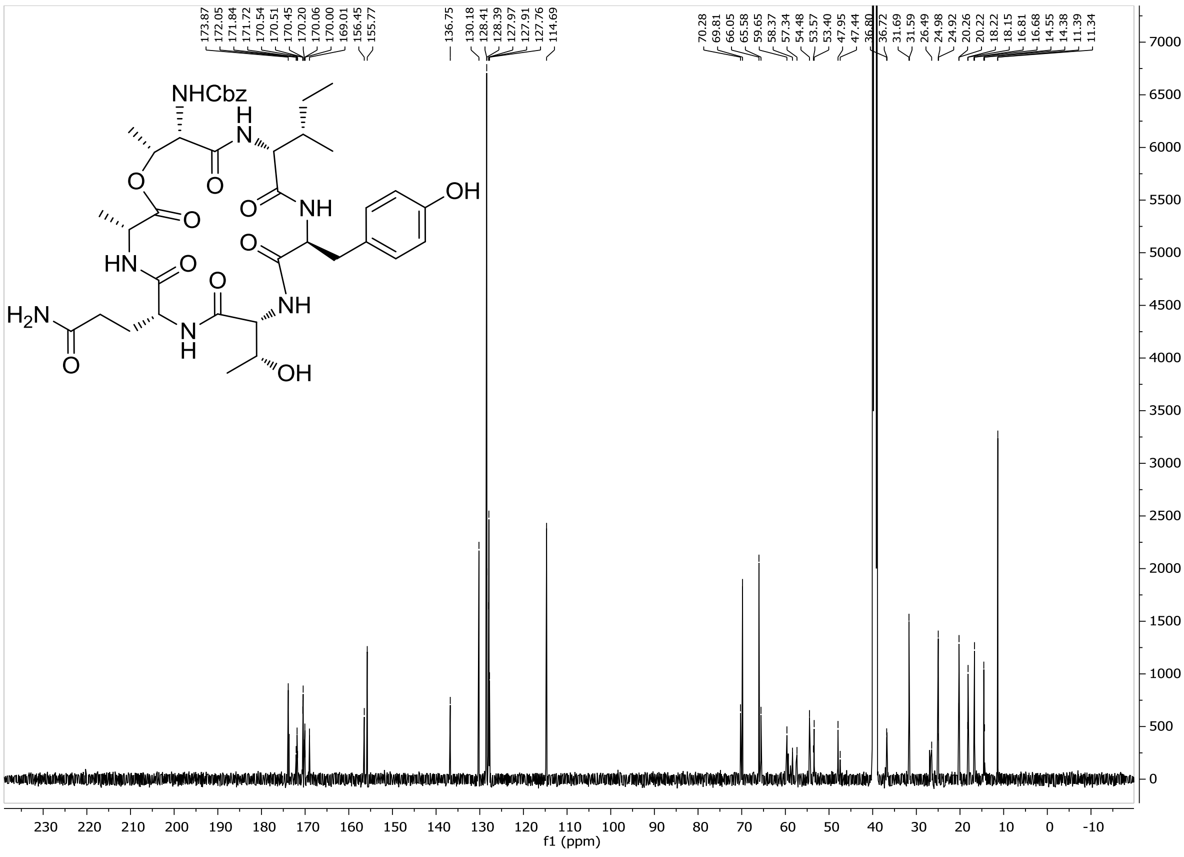

**Figure S8: $^{1}$H-NMR (600 MHz, DMSO-$d_6$) and $^{13}$C-NMR (150 MHz, DMSO-$d_6$) of compound 19**

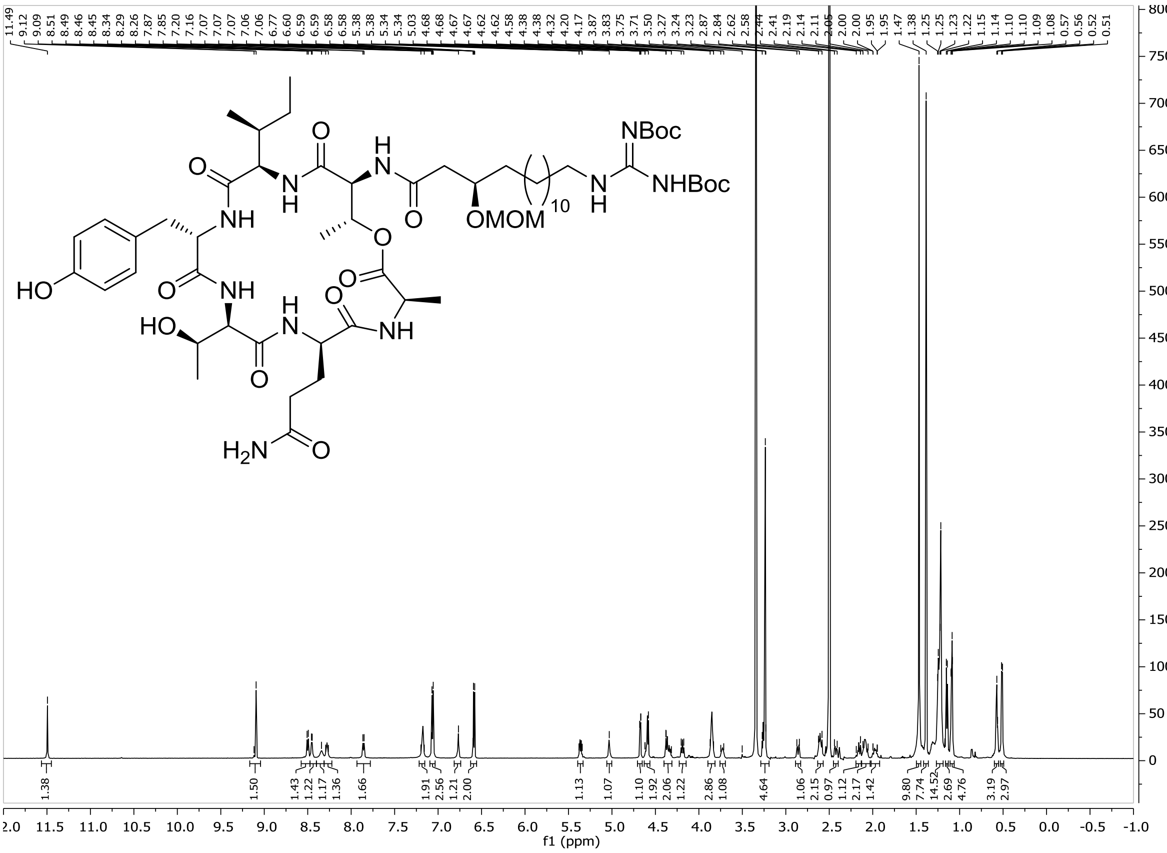

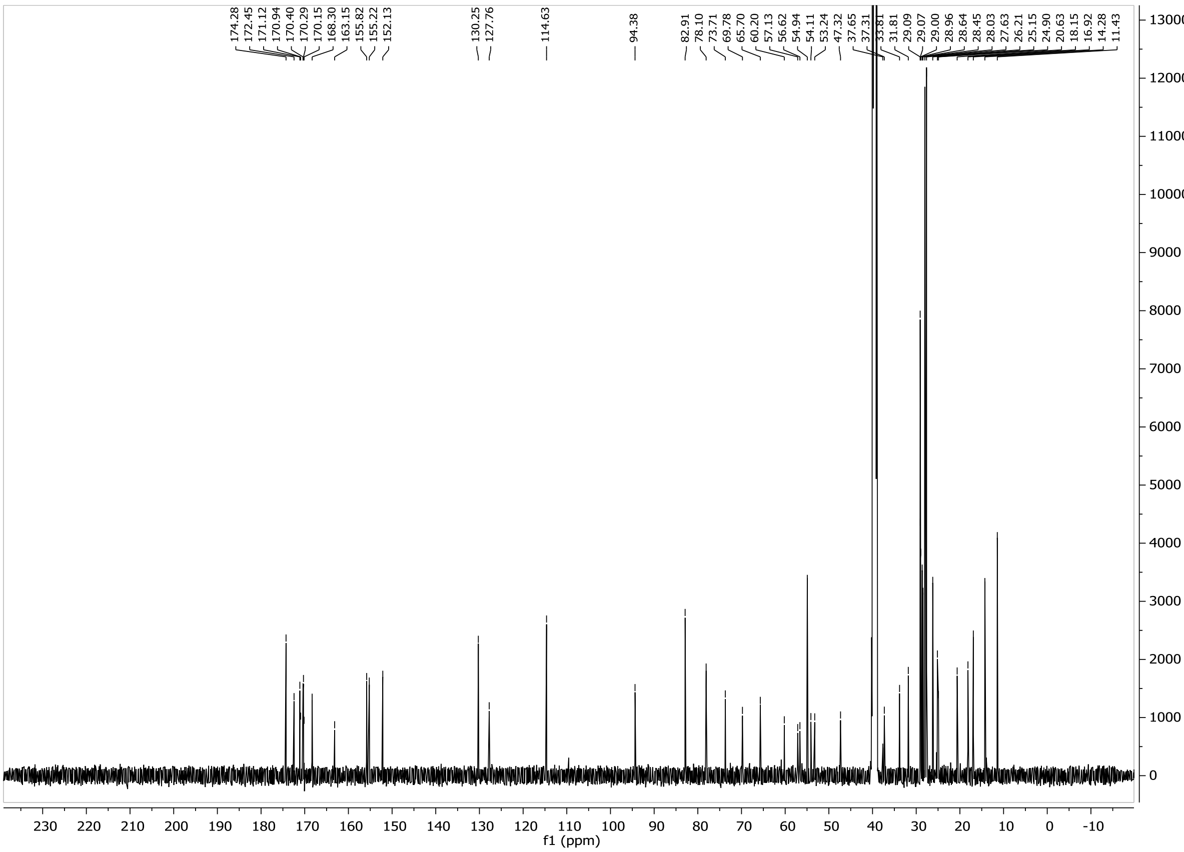

**Figure S9: $^{1}$H-NMR (600 MHz, DMSO-$d_6$) and $^{13}$C-NMR (150 MHz, DMSO-$d_6$) of compound 24**

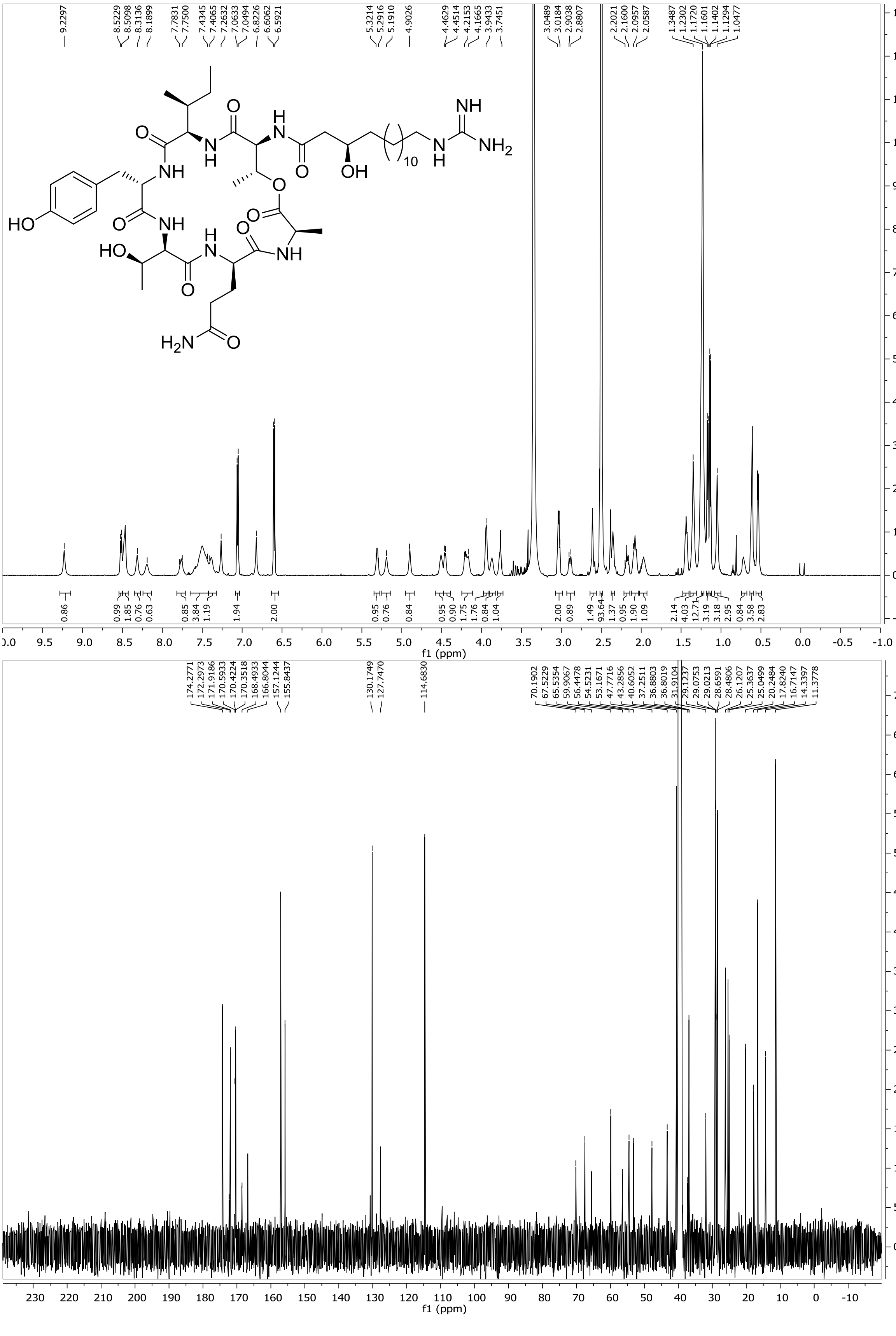


**Figure S10: $^{1}$H-NMR (600 MHz, DMSO-$d_6$) and $^{13}$C-NMR (150 MHz, DMSO-$d_6$) of synthetic compound 1**

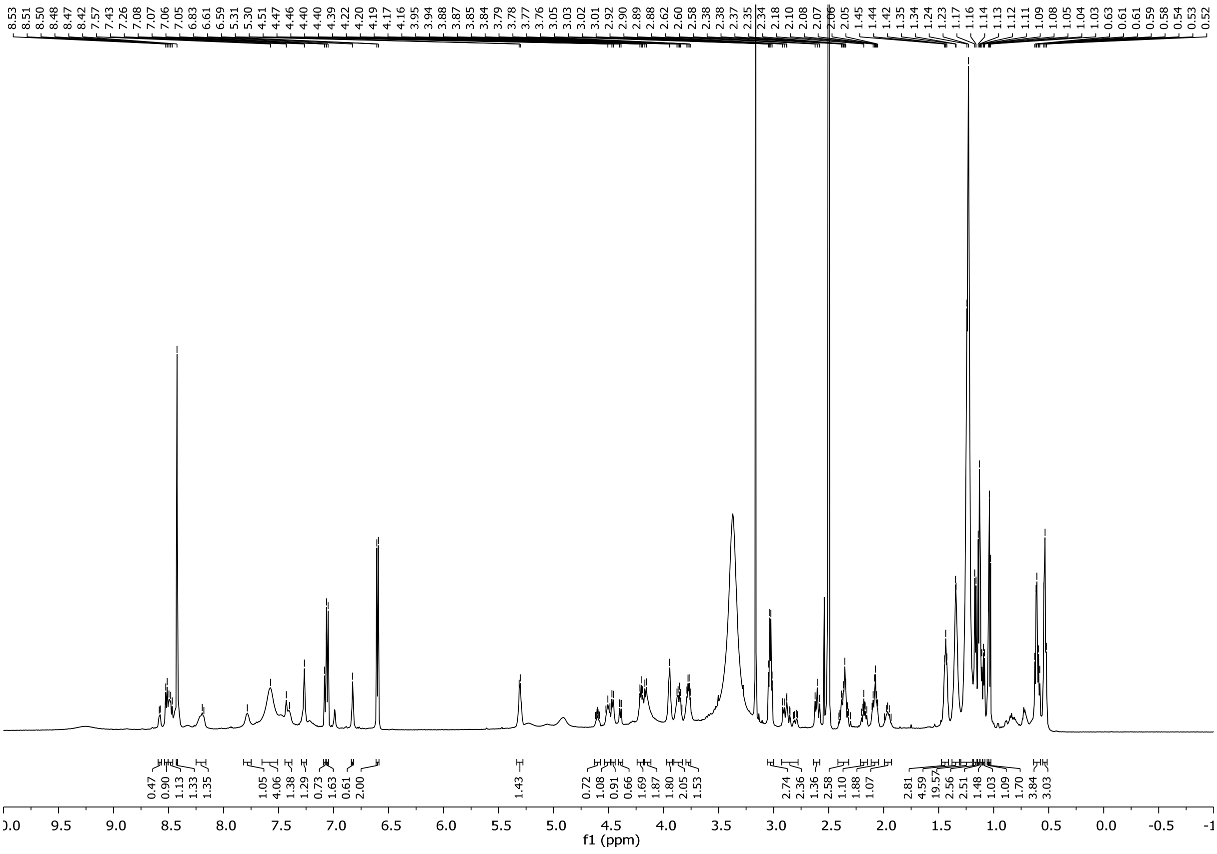


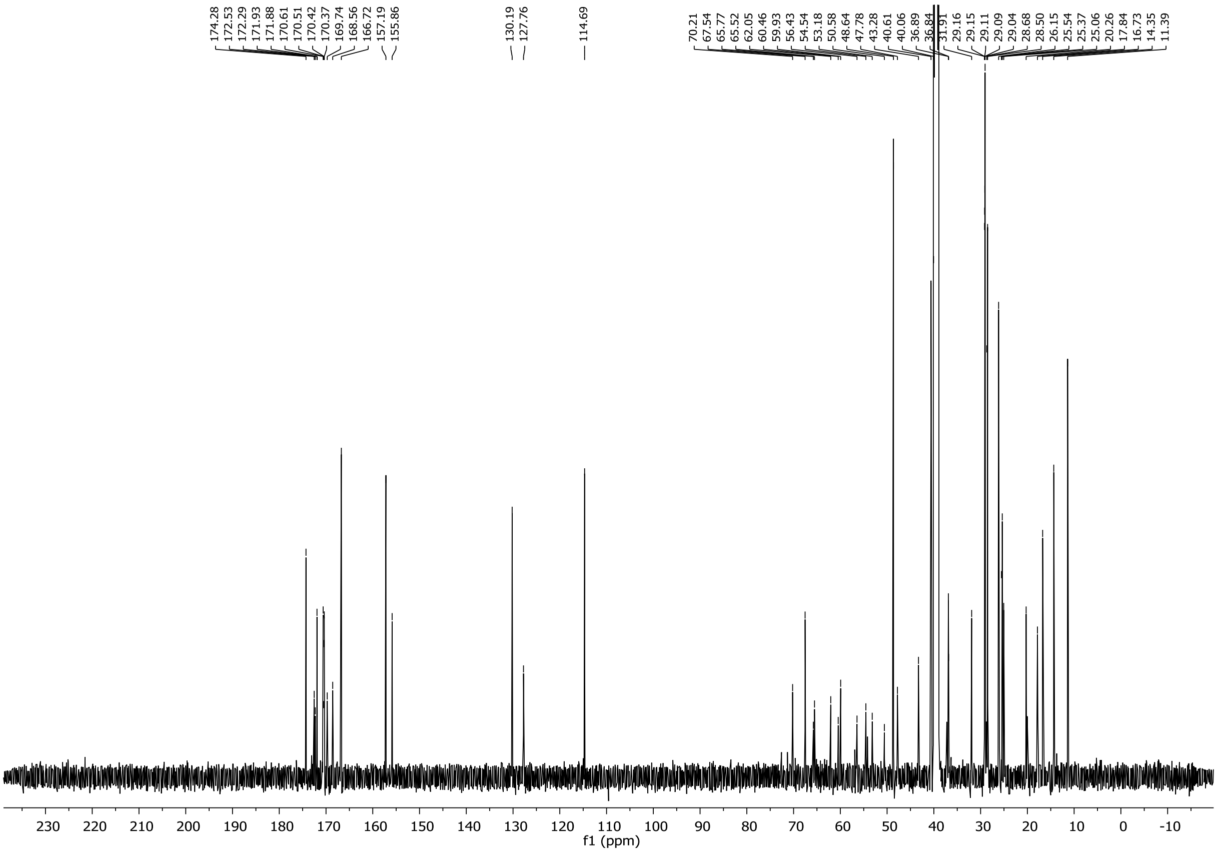


**Figure S11: $^{1}$H-NMR (600 MHz, DMSO-$d_6$) and $^{13}$C-NMR (150 MHz, DMSO-$d_6$) of isolated compound mixture**

## IV) 2D-NMR Spectra of selected compounds

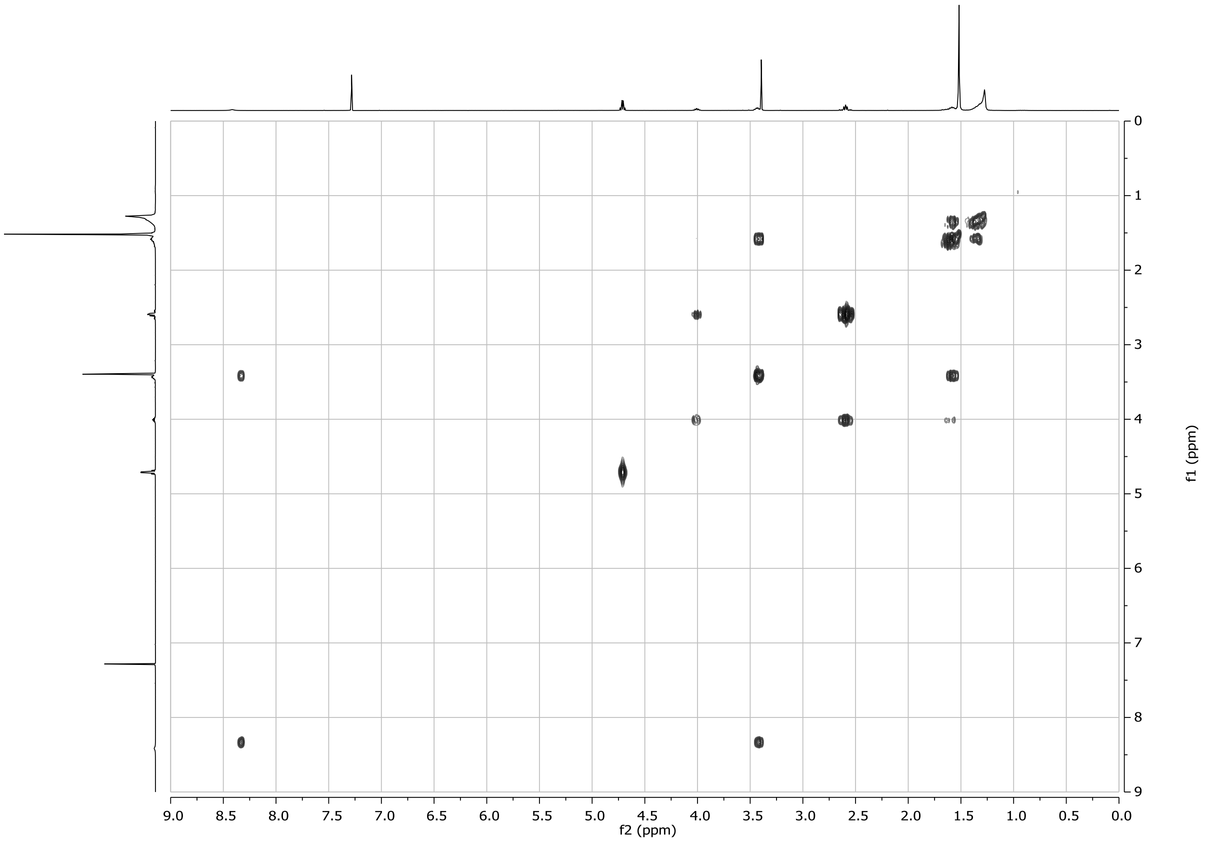


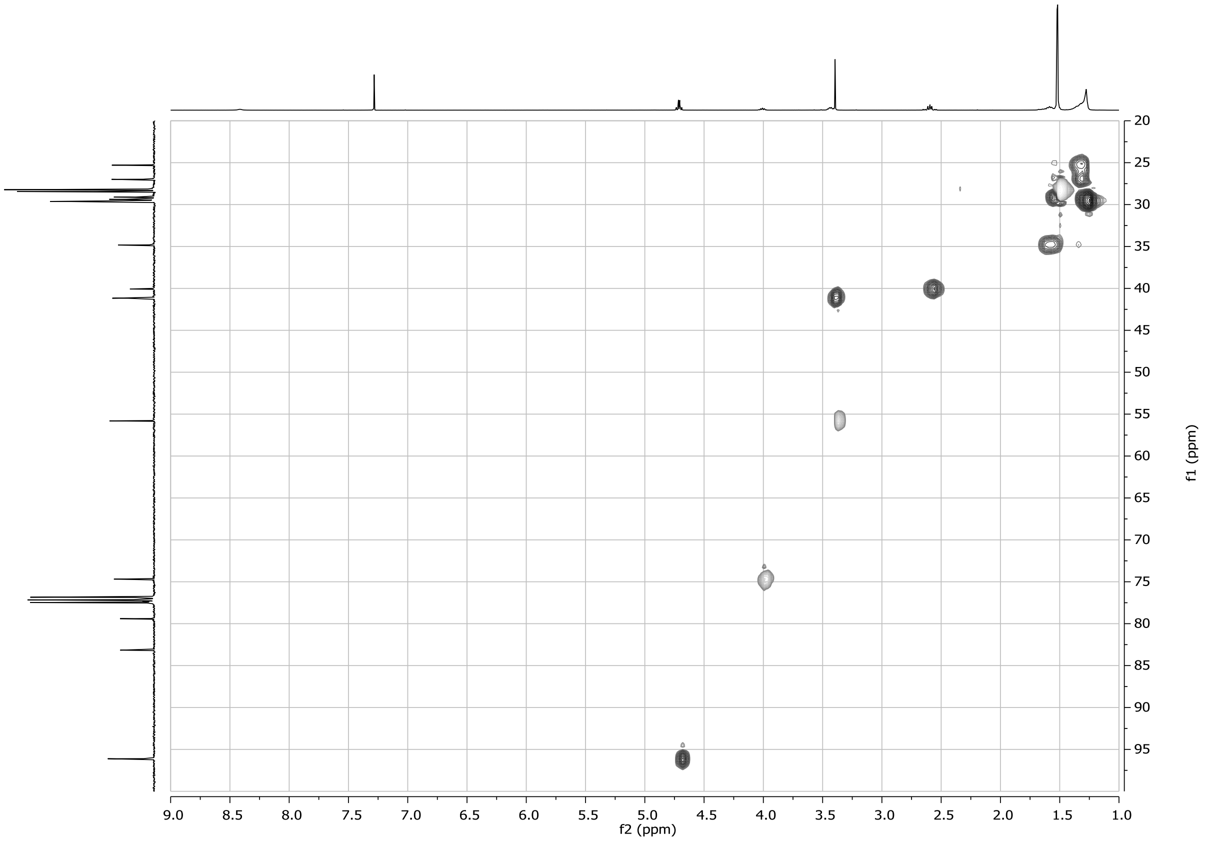


**Figure S12: COSY (400 MHz, $CDCl_3$)and HSQC (400/101 MHz, $CDCl_3$) of compound 3**

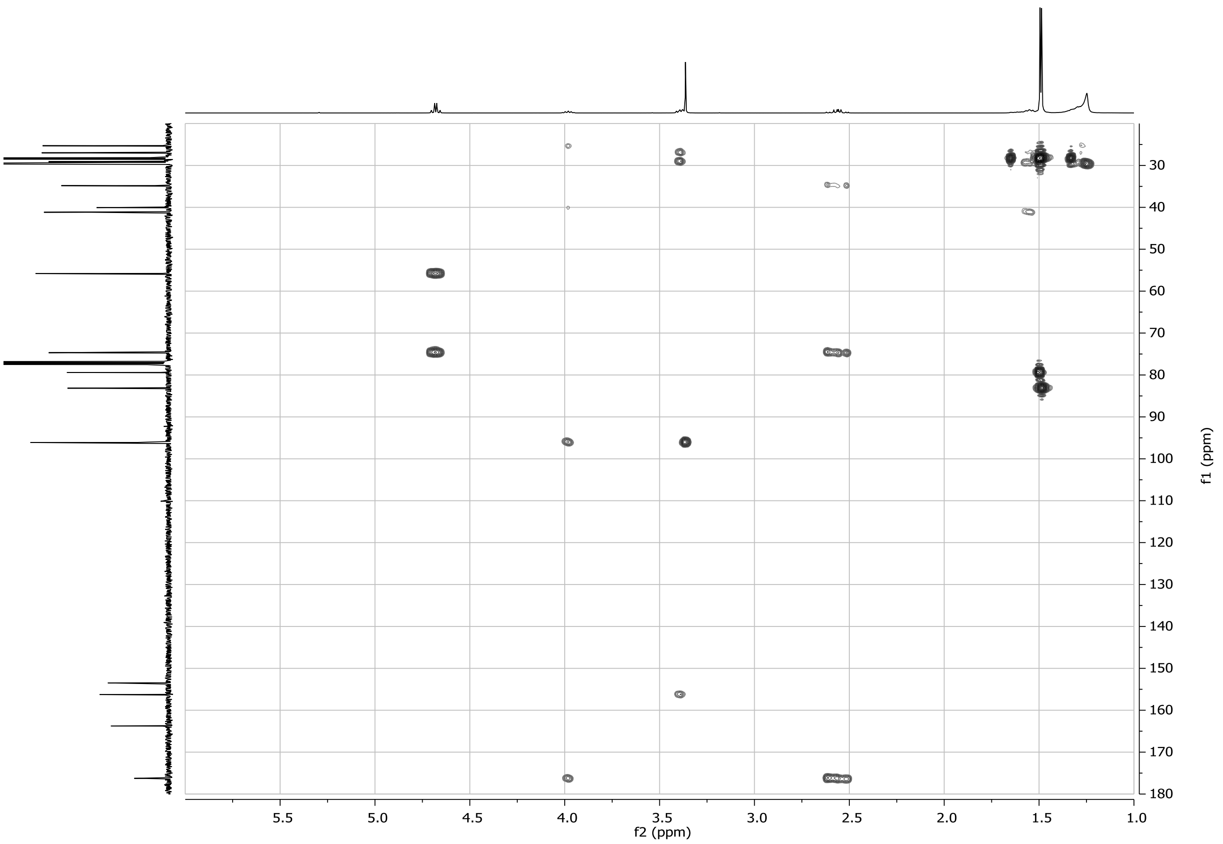


**Figure S13: HMBC (400/101 MHz, $CDCl_3$) of compound 3**

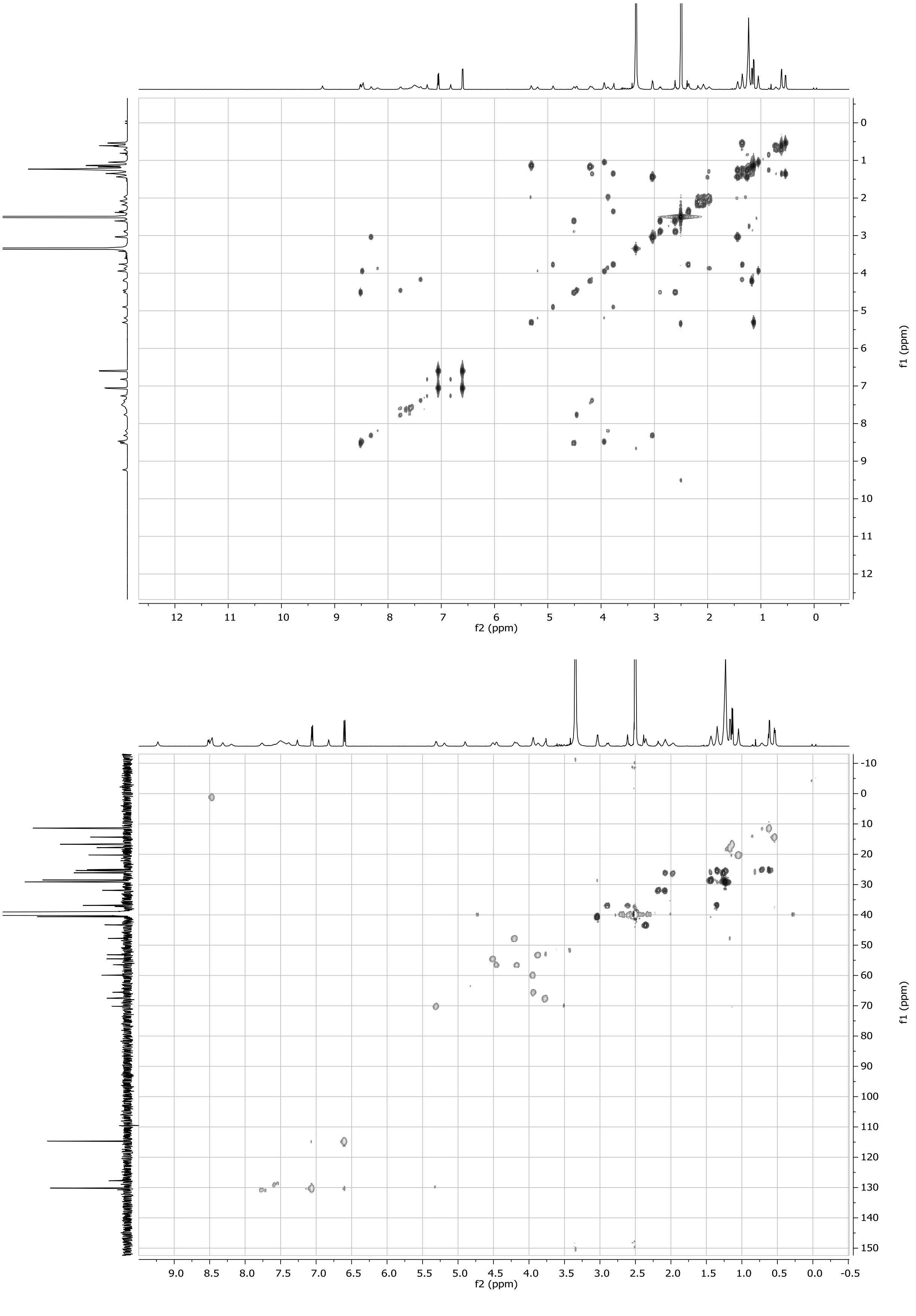


**Figure S14: COSY (600 MHz, DMSO-$d_6$) and HSQC (600/150 MHz, DMSO-$d_6$) of synthetic compound 1**

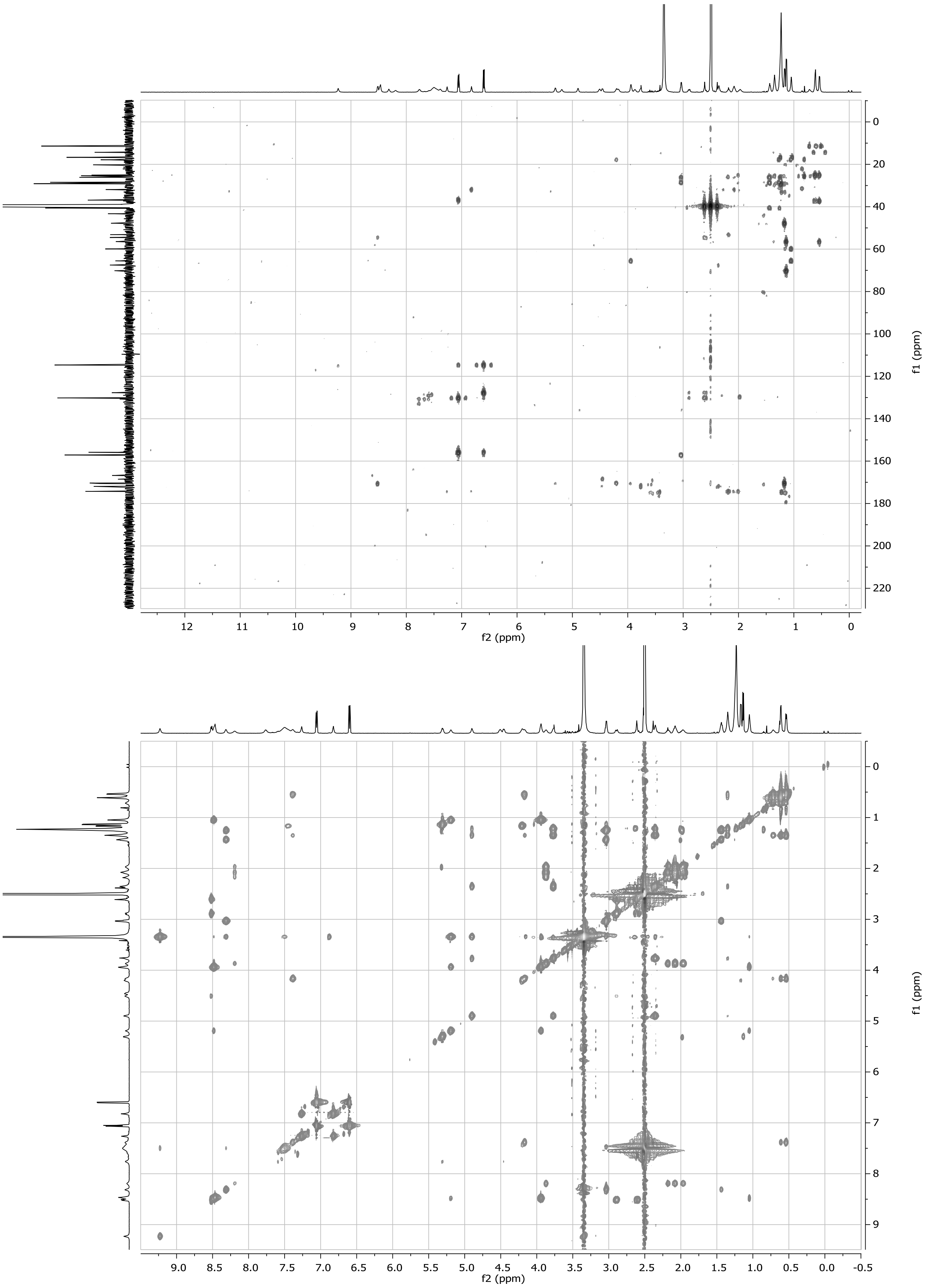


**Figure S15: HMBC (600/150 MHz, DMSO-$d_6$) and TOCSY (600 MHz, DMSO-$d_6$) of synthetic compound 1**

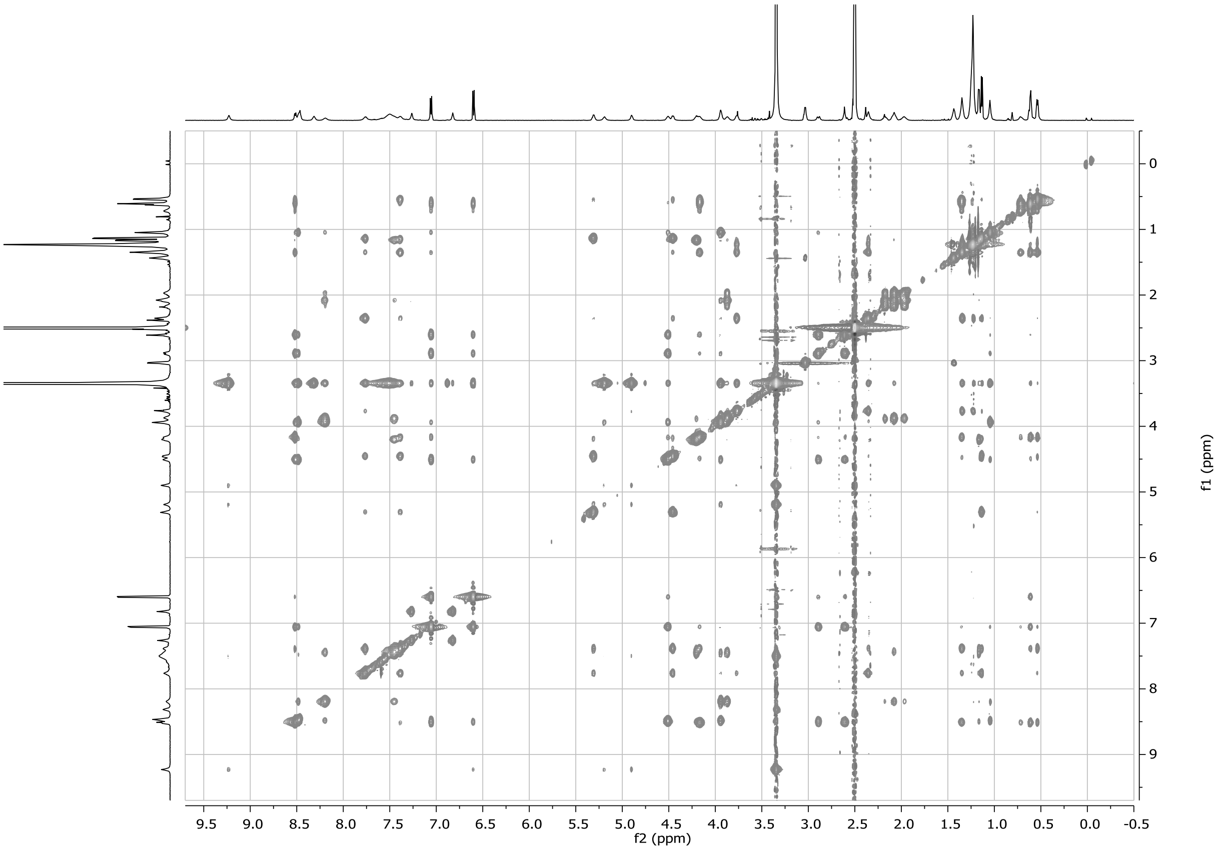


**Figure S16: NOESY (600 MHz, DMSO-$d_6$) of synthetic compound 1**

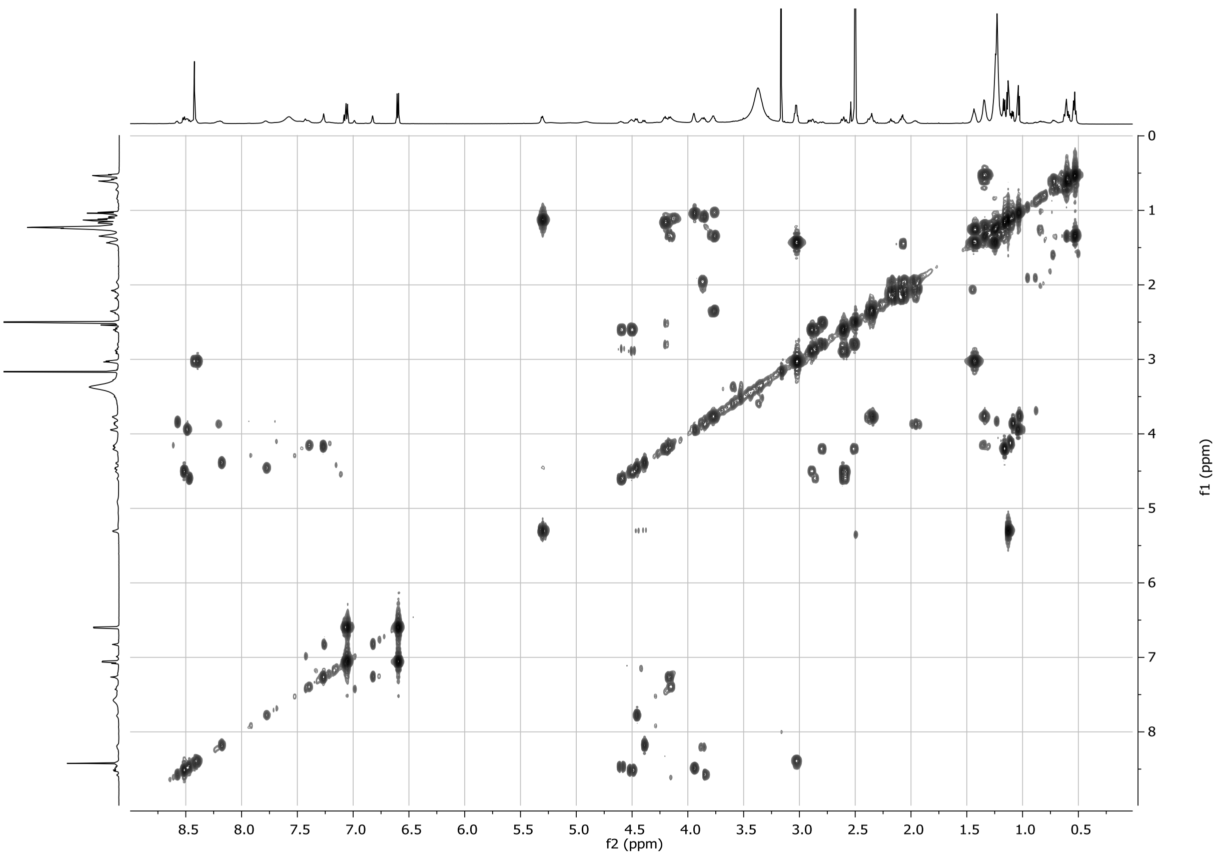


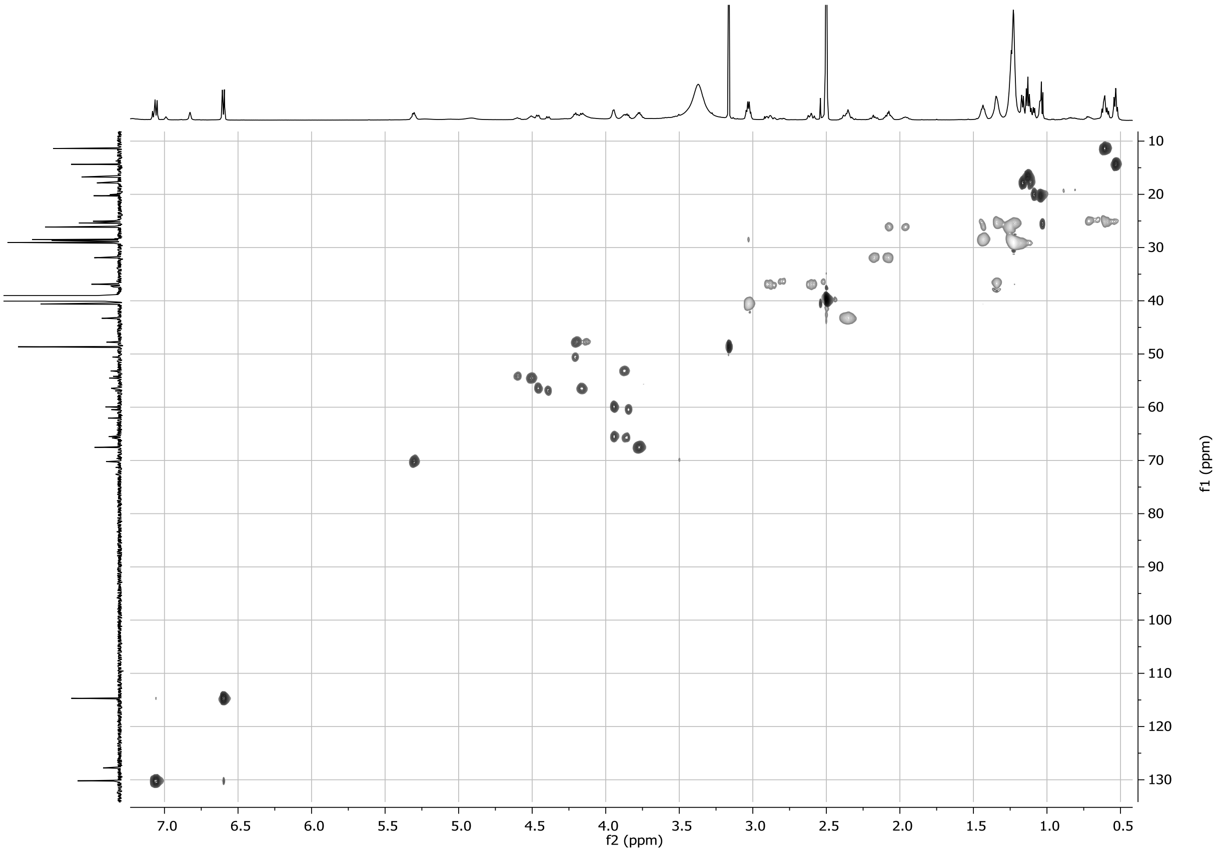


**Figure S17: COSY (600 MHz, DMSO-$d_6$) and HSQC (600/150 MHz, DMSO-$d_6$) of isolated compound mixture**

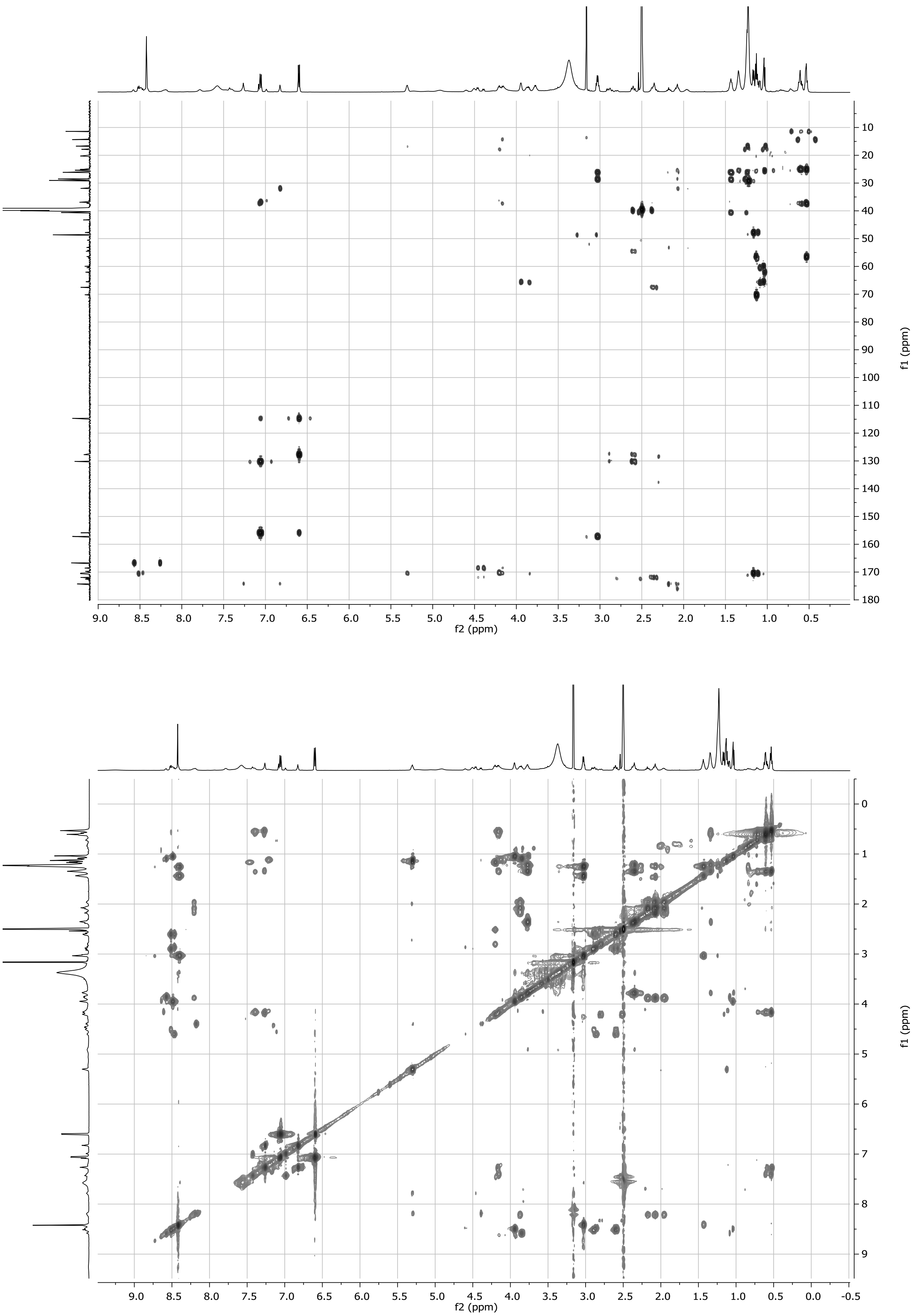


**Figure S18: HMBC (600/150 MHz, DMSO-$d_6$) and TOCSY (600 MHz, DMSO-$d_6$) of isolated compound mixture**

## V) Mosher Analysis Spectra and selected carbon shifts of cyclopeptide 19

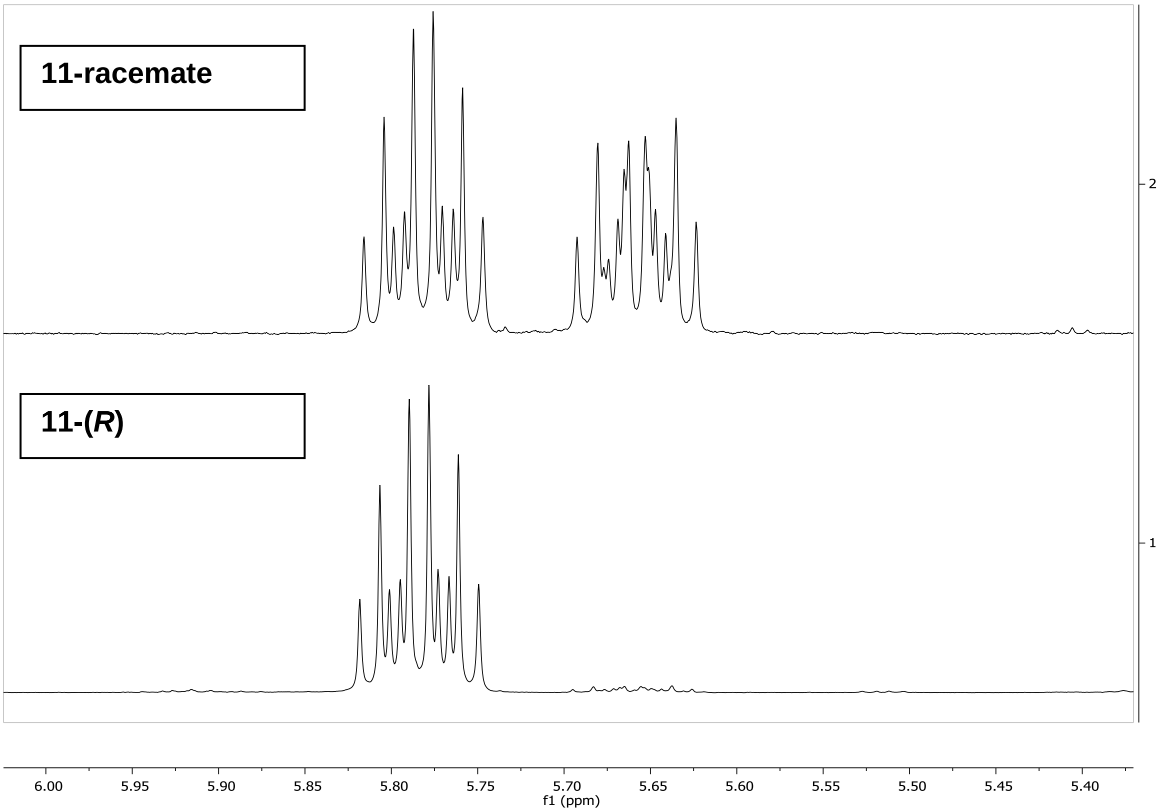


**Figure S19: $^{1}H$-NMR (600 MHz, $CDCl_3$) spectra from Mosher Analysis of (*R*)-11 with *R*-MTPA, top: racemate, bottom: (*R*)-enantiomer (94% ee)**

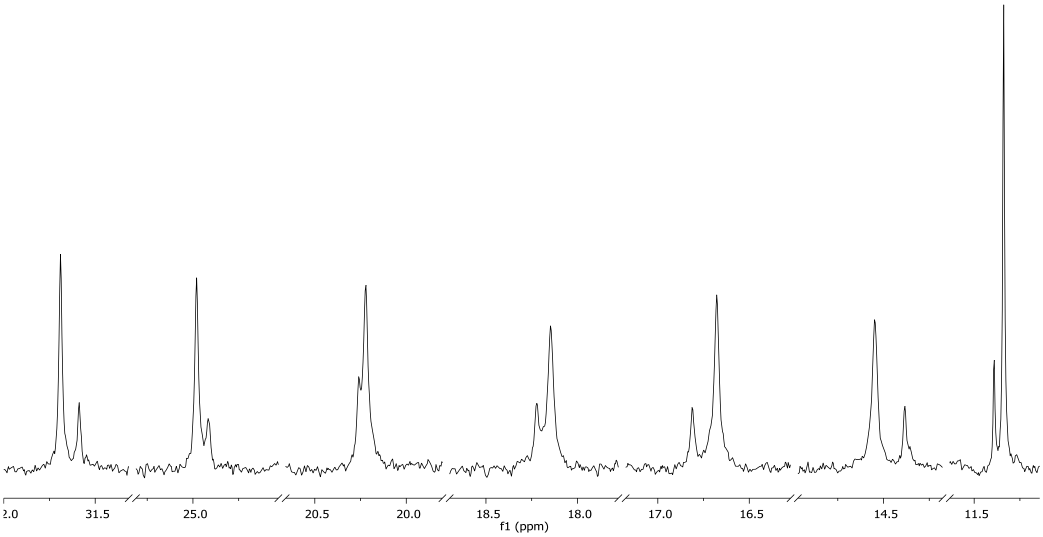


**Figure S20: selected $^{13}C$-NMR shifts showing the double signal set**

## VI) Shift table and $^{13}$C-NMR spectra comparison of 1, synthetic vs. natural

| 150 MHz DMSO 294 K, in ppm | | | | | | | | | |
|---|---|---|---|---|---|---|---|---|---|
| | | δC natural | δC synthetic | δΔ | | | δC natural | δC synthetic | δΔ |
| **Thr** | 1 | 168.6 | 168.5 | -0.1 | **allo-Ile** | 26 | 170.4 | 170.4 | 0 |
| | 2 | 56.5 | 56.5 | 0 | | 27 | 56.5 | 56.5 | 0 |
| | 3 | 70.2 | 70.2 | 0 | | 28 | 37.2 | 37.2 | 0 |
| | 4 | 16.7 | 16.7 | 0 | | 29 | 25.1 | 25.1 | 0 |
| **Ala** | 5 | 170.4 | 170.4 | 0 | | 30 | 14.4 | 14.4 | 0 |
| | 6 | 47.8 | 47.8 | 0 | | 31 | 11.4 | 11.4 | 0 |
| | 7 | 17.8 | 17.8 | 0 | **GHPD** | 32 | 171.9 | 171.9 | 0 |
| **Gln** | 8 | 170.4 | 170.4 | 0 | | 33 | 43.3 | 43.3 | 0 |
| | 9 | 53.2 | 53.2 | 0 | | 34 | 67.6 | 67.5 | -0.1 |
| | 10 | 26.2 | 26.1 | -0.1 | | 35 | 36.9 | 36.9 | 0 |
| | 11 | 32 | 32 | 0 | | 36-44 | 28.7-29.2 | 28.7-29.2 | |
| | 12 | 174.3 | 174.3 | 0 | | 45 | 28.5 | 28.5 | 0 |
| **allo-Thr** | 13 | 170.6 | 170.6 | 0 | | 46 | 40.6 | 40.6 | 0 |
| | 14 | 59.9 | 59.9 | 0 | | 47 | 157.2 | 157.1 | -0.1 |
| | 15 | 65.6 | 65.6 | 0 | | | | | |
| | 16 | 20.1 | 20.2 | 0.1 | | | | | |
| **Tyr** | 17 | 166.7 | 166.8 | 0.1 | | | | | |
| | 18 | 54.6 | 54.5 | -0.1 | | | | | |
| | 19 | 36.9 | 36.9 | 0 | | | | | |
| | 20 | 127.8 | 127.7 | -0.1 | | | | | |
| | 21. 25 | 130.2 | 130.2 | 0 | | | | | |
| | 22. 24 | 114.7 | 114.7 | 0 | | | | | |
| | 23 | 155.9 | 155.9 | 0 | | | | | |

**Table S1: Shifttable of the $^{13}$C-NMR data comparison synthetic vs. natural compound 1**

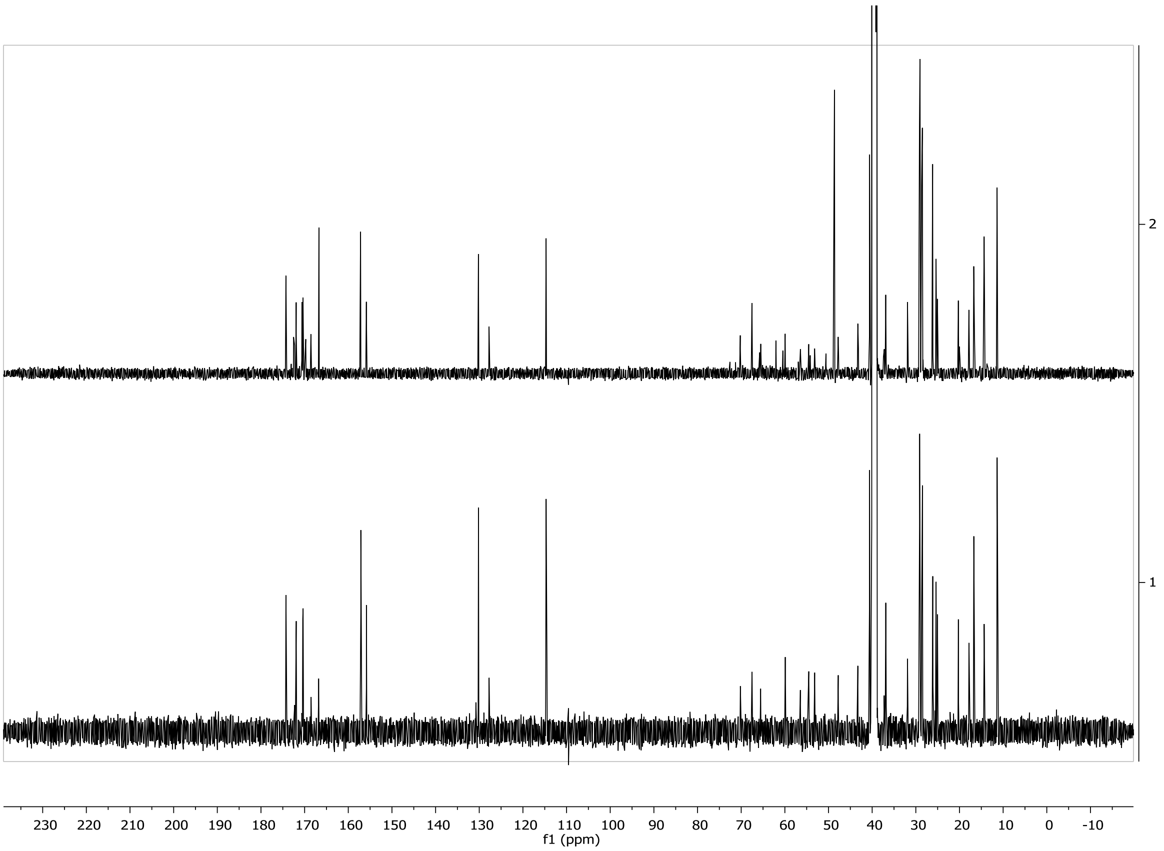


**Figure S21: top: $^{13}$C-NMR (150 MHz, DMSO-$d_6$) of the isolated compound mixture**
**bottom: $^{13}$C-NMR (150 MHz, DMSO-$d_6$) of synthetic compound 1**

# VII) References


1. G. R. Fulmer, A. J. M. Miller, N. H. Sherden, H. E. Gottlieb, A. Nudelman, B. M. Stoltz, J. E. Bercaw and K. I. Goldberg, *Organometallics*, **2010**, 29, 2176-2179.
2. G. Breton, J. Shugart, C. Hughey, B. Conrad and S. Perala, *Molecules*, **2001**, 6, 655.
3. Y. Koseki, H. Yamada and T. Usuki, *Tetrahedron: Asymmetry*, **2011**, 22, 580-586.
4. M. Stawikowski and P. Cudic, *Tetrahedron Lett.*, **2006**, 47, 8587-8590.
5. N. Bionda, M. Stawikowski, R. Stawikowska, M. Cudic, F. López-Vallejo, D. Treitl, J. Medina-Franco and P. Cudic, *ChemMedChem*, **2012**, 7, 871-882.
